# Electric vehicles charging stations load forecasting based on hybrid XGBoost-BiLSTM model

**DOI:** 10.1038/s41598-025-29739-z

**Published:** 2026-01-02

**Authors:** Hany S. E. Mansour, Amira S. Mohamed, M. Abdel-Aziz

**Affiliations:** 1 Electrical Engineering Department, Faculty of Engineering, Suez Canal University, Ismailia, 41522 Egypt; 2 Department of Basic Sciences, Faculty of Computer and Information Sciences, Ain Shams University, Cairo, 11566 Egypt; 3 Department of Artificial Intelligence Engineering, Faculty of Computer Science and Engineering, King Salman International University (KSIU), South Sinai, 46511 Egypt

**Keywords:** Artificial intelligence, Electric vehicles, Electric vehicle charging load forecasting, Hybrid forecasting model, Stacking ensemble learning, XGBoost, BiLSTM, Energy science and technology, Engineering, Mathematics and computing

## Abstract

Accurate load forecasting for Electric Vehicle Charging Stations (EVCS) is critical for optimizing energy management and ensuring grid stability amid growing electric vehicle adoption. This study investigates short-term, hourly load forecasting at the station level using a hybrid XGBoost–BiLSTM stacking model (Hybrid 3) with an XGBoost meta-learner. From the Adaptive Charging Network (ACN–Caltech) dataset (April 25, 2018–September 13, 2021), 31,424 raw charging sessions were preprocessed, yielding 14,496 cleaned sessions for modeling. These were split into 80% training and 20% testing sets using a fixed random seed (42) for reproducibility. Hybrid 3 was benchmarked against 24 alternative models spanning statistical (e.g., Persistence, SARIMAX), machine learning (e.g., XGBoost, LightGBM), deep learning (e.g., BiLSTM, CNN), and ensemble methods. On cleaned data, Hybrid 3 achieved an MAE of 2.6870 kWh and R^2^ = 0.6395—a 3.4% improvement over standalone BiLSTM—while slightly underperforming the top Boosting ensemble (XGBoost + BiLSTM + LightGBM). Robustness was confirmed via five-fold walk-forward validation (mean MAE = 2.5351 kWh, SD = 1.2885). Cross-site evaluation on an independent synthetic dataset (n = 1,965,239 sessions) showed reduced generalization, highlighting site-specific temporal patterns. One-Way ANOVA (p = 0.2073, η^2^ = 0.2062) indicated no statistically significant but practically relevant differences among top models. Feature importance analysis identified log-transformed charging duration (importance = 0.376) as the dominant predictor, aligning with real-world EV behavior. Overall, Hybrid 3 balances accuracy and complexity effectively, though gradient-boosting ensembles remain preferable for scalable, real-time EVCS forecasting.

## Introduction

Modern power systems integrate generation, transmission, and distribution to ensure efficient, reliable electricity delivery. These systems leverage advanced technologies to manage power flow, aiming for sustainable, cost-effective supply amid growing demand while minimizing environmental impact^1^. Accurate load forecasting is critical for grid operations, supporting short-term decisions (e.g., unit commitment, dispatch) and long-term planning (e.g., infrastructure expansion). Inaccurate forecasts risk increased costs, system instability, or blackouts^2^. The rapid rise in Electric Vehicle (EV) adoption has driven widespread charging station deployment, fueled by efforts to reduce fossil fuel reliance^3^. The European Union targets 750,000 public charging stations by 2025 (Alternative Fuels Infrastructure Regulation), the United States aims for 500,000 by 2030 (Bipartisan Infrastructure Law), and China leads with over 1.4 million public charging points, including high-speed chargers along major corridors. However, volatile EV charging demand, driven by unpredictable user behaviors, strains grid infrastructure^4^. Precise short-term, hourly, station-level load forecasting is essential for optimizing power consumption, resource allocation, and ensuring grid stability^5,6^. EV load forecasting methods include physically based and data-driven approaches. Physically based methods, like trip chain analysis^7^, Monte Carlo simulations^8^, and Markov decision processes^9^, are computationally intensive and less scalable^10^. Data-driven approaches, including statistical and AI-based techniques, are more efficient^11^. Statistical models, such as Autoregressive Integrated Moving Average (ARIMA) for EV loads^12^ or seasonal ARIMA for station profiles^13^, require minimal data but struggle with complex patterns. An adaptive Multilayer Perceptron (MLP) was developed for vehicle-to-grid scheduling^14^. Machine learning (ML) methods, like Support Vector Machines, Random Forests, and eXtreme Gradient Boosting (XGBoost)^15^, excel at nonlinear relationships but require extensive feature engineering and risk overfitting^16^. Deep learning (DL) approaches, including Convolutional Neural Networks (CNN), Long Short-Term Memory (LSTM), and Gated Recurrent Units, capture temporal dependencies^17^. Multivariate LSTM models with external factors (e.g., temperature, humidity) outperform univariate models^18^, while dynamic learning rates enhance forecasting at 15-min intervals^19^. Studies comparing LSTM, Bidirectional LSTM (BiLSTM), and CNN-LSTM highlight external factor impacts^4^. Advanced techniques, like adaptive proximal policy optimization^20^, Bayesian inference^21^, and hybrid Convolutional LSTM^22^, further improve accuracy.

Recent stacking-based ensembles have shown promise in energy forecasting by fusing diverse learners to mitigate overfitting and enhance generalization. For instance, a stack-based ensemble with meta-neural networks (SEFMNN) integrates ANN, RF, and SVM for solar irradiance prediction, achieving R^2^ > 0.99 while balancing computational efficiency^23^. Similarly, transformer-infused recurrent networks (TIR), combining BiLSTM encoders with GRU decoders and attention mechanisms, optimize short-term irradiance forecasts across regions like Munich and Texas, yielding RMSE as low as 0.014 with superior handling of meteorological variability^24^. Control-oriented advances, such as reinforcement learning (RL) frameworks like PPO and Meta-RL, enable dynamic adaptation in solar energy management, reducing operational costs by smoothing ramps and optimizing storage under uncertainty^25^. In contrast, our XGBoost-BiLSTM stacking (Hybrid 3) tailors these principles to EV charging dynamics, leveraging XGBoost as a meta-learner to ensemble tree-based nonlinearity with bidirectional temporal modeling—addressing EV-specific challenges like session volatility absent in solar data—while requiring less meteorological input than TIR or RL hybrids. Single ML or DL models are often inadequate for capturing both nonlinear patterns and temporal dependencies in EV charging data. XGBoost excels at modeling complex feature interactions but struggles with sequential dependencies, whereas BiLSTM effectively captures bidirectional temporal patterns but may miss intricate nonlinear relationships. Hybrid models combining ML and DL are increasingly recognized for addressing these shortcomings, yet their application to EV load forecasting remains underexplored.

A novel hybrid XGBoost–BiLSTM stacking ensemble (Hybrid 3) is proposed for short-term, hourly, station-level electric vehicle (EV) charging load forecasting. The model integrates XGBoost’s gradient-boosted decision trees with BiLSTM’s bidirectional temporal modeling, unified through an XGBoost meta-learner that learns optimal nonlinear combinations of the base learners’ predictions.

The proposed framework is evaluated using the Adaptive Charging Network (ACN) dataset, comprising 31,424 charging sessions collected from the Caltech mixed-use facility (April 2018–September 2021). Comprehensive benchmarking was performed against 24 baseline models spanning statistical (Persistence, Seasonal Naïve, SARIMAX, Prophet), machine learning (XGBoost, LightGBM), deep learning (CNN, TCN, Transformer, BiLSTM), and ensemble families (boosting, bagging, stacking, and weighted combinations).

On the cleaned dataset, Hybrid 3 achieved an MAE of 2.687 kWh and R^2^ = 0.6395, outperforming the standalone BiLSTM by 3.4%, and ranking among the top models while balancing accuracy, interpretability, and complexity. On the original (outlier-retained) dataset, Hybrid 3 maintained strong performance (MAE = 3.543 kWh, R^2^ = 0.5285), confirming robustness to noisy inputs.

Temporal robustness was validated through five-fold walk-forward validation, where Hybrid 3 achieved the lowest mean MAE (2.54 kWh) and highest mean R^2^ (0.63) across folds. One-way ANOVA (F = 1.624, p = 0.2073) showed no statistically significant differences among top models; however, the large effect size (η^2^ = 0.2062) indicated practical improvement.

Feature importance analysis identified charging duration (log-transformed) as the most influential predictor (importance = 0.376), emphasizing the dominant role of session length in determining delivered energy. The ablation study confirmed the critical contribution of each component: removing feature engineering (+ 13.7% MAE degradation), removing base learners (+ 18.8%), and removing the meta-learner (+ 29.2%), underscoring the stacking layer as the main performance driver.

External generalization was assessed using a cross-site synthetic dataset (~ 1.96 M sessions), where Hybrid 3 exhibited transferable but sensitivity-limited performance (MAE = 4.16 kWh, R^2^ = 0.01), while bagging ensembles achieved the best transfer accuracy (MAE = 3.08 kWh, R^2^ = 0.36). This study extends prior hybrid forecasting research (e.g., CNN-LSTM for smart building energy^26^) by introducing a stacking-enhanced XGBoost–BiLSTM framework specifically tailored for EV charging station load dynamics.

### Major contributions

- Development of a hybrid XGBoost–BiLSTM stacking ensemble (Hybrid 3) that integrates gradient boosting and bidirectional sequence learning for short-term EV charging load forecasting.
- Design of a comprehensive benchmarking framework encompassing 24 baseline models across statistical, machine learning, deep learning, and ensemble families.
- Implementation of walk-forward validation to ensure temporal robustness and realistic performance assessment under non-stationary conditions.
- Execution of a detailed ablation study to quantify the contribution of each model component, highlighting the role of stacking and feature engineering in enhancing predictive performance.
- Integration of cross-site validation to evaluate model generalization and external validity under domain shift scenarios.

**Fig. 1:**
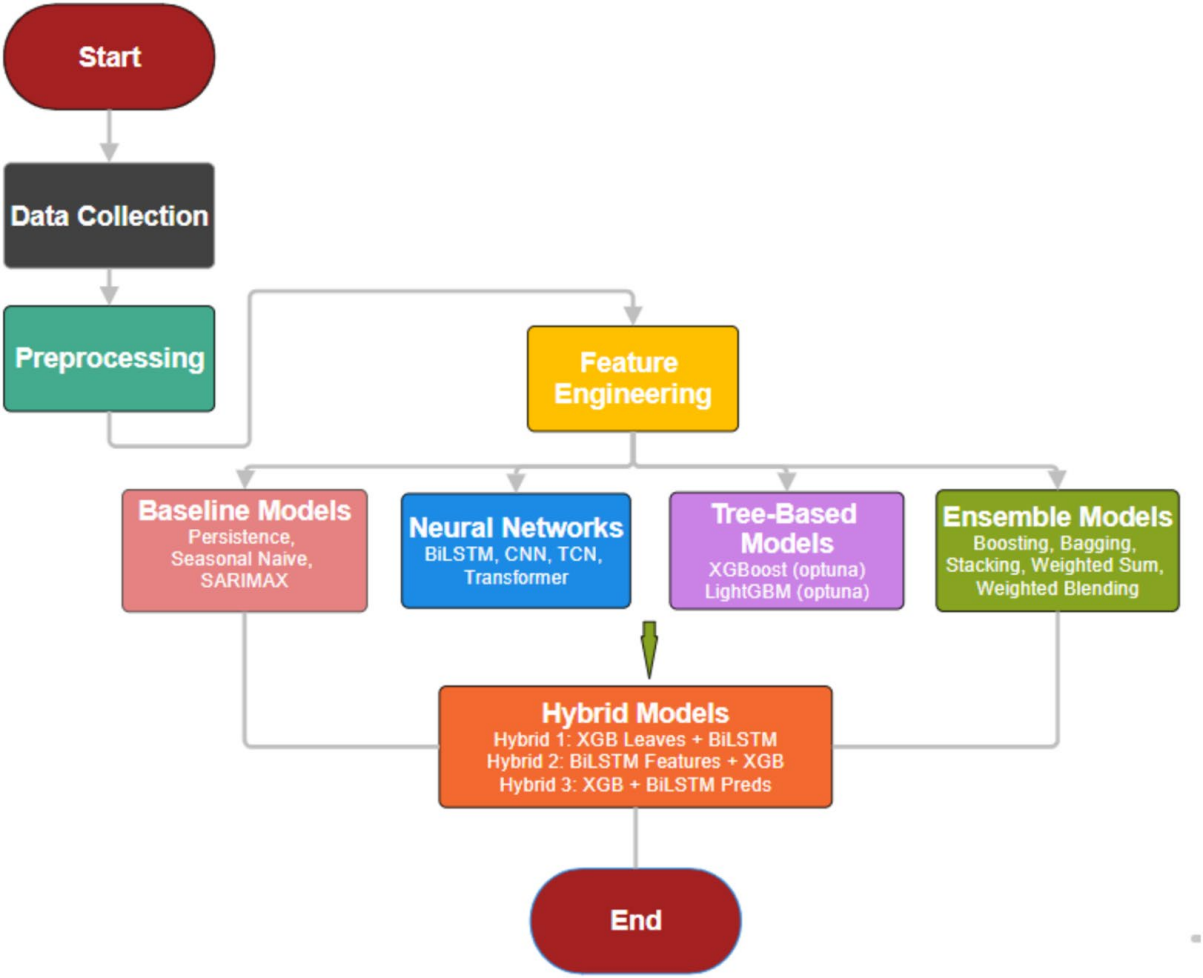
Research methodology.

Overall, this work introduces a stacking-enhanced hybrid architecture tailored to EV charging dynamics, combining the interpretability of gradient boosting with the sequence modeling capability of BiLSTM. It establishes a robust foundation for data-driven EV energy forecasting, supporting effective charging network management and grid integration. Figure 1 presents the complete research methodology.

## Methodology

### Dataset

This study utilizes the Adaptive Charging Network (ACN) dataset^27^, a comprehensive record of workplace EV charging sessions at a mixed-use parking facility on the Caltech campus. The dataset includes individual session characteristics and high-resolution time series data capturing power output for each charging unit. Key variables include EV arrival and departure timestamps, energy requested, and actual energy delivered (kWhDelivered) per session. The analysis covers data from 55 charging stations, primarily accessible to Caltech employees but also available for public use, including gym patrons. This mixed-use context makes the ACN dataset representative of both workplace and public charging patterns. The dataset spans 1329 days, from April 25, 2018, to September 13, 2021, comprising 31,424 charging sessions used for model training and evaluation. A summary of key data fields analyzed is presented in Table 1.

**Table 1:** ACN data field and description.

Field	Description	Data type	Unit / format
_id	Unique identifier for the database record	String	–
userInputs	Optional user-provided metadata or session notes	String / Null	–
sessionID	Unique identifier for the charging session	String	–
stationID	Identifier for the EV supply equipment	String	–
spaceID	Identifier of the parking space	String	–
siteID	Identifier of the site where the session took place	String	–
clusterID	Group identifier for related stations/sites	String	–
connectionTime	Timestamp when the vehicle was plugged in	Datetime	ISO 8601
disconnectTime	Timestamp when the vehicle was unplugged	Datetime	ISO 8601
kWhDelivered	Total energy delivered during the session	Float	kWh
doneChargingTime	Timestamp when charging completed	Datetime	ISO 8601
timezone	Local timezone of the site	String	Olson identifier
userID	Anonymized identifier for the user	String / Null	–

#### Dataset analysis

**Fig. 2:**
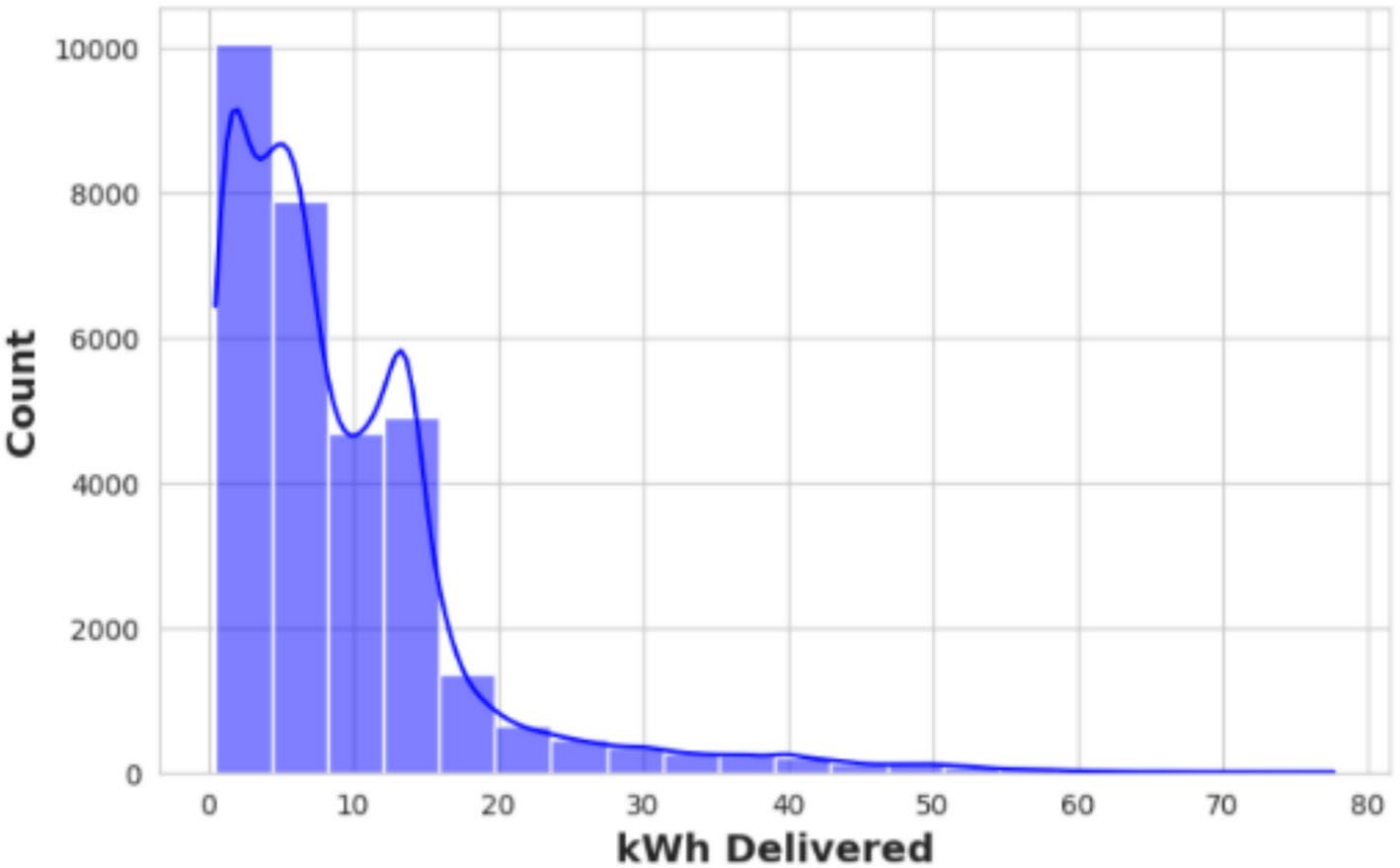
Distribution of delivered energy.

To gain a clearer understanding of the characteristics within these datasets, a concise exploratory analysis was performed. Figure 2 presents a histogram depicting the distribution of energy delivered during individual charging sessions, overlaid with a kernel density estimate (KDE). The distribution exhibits a marked right skew, with frequencies sharply decreasing as the amount of energy delivered increases. This suggests that most charging sessions involve relatively small energy transfers. Notably, there is an uptick in frequencies between 2 and 10 kWh, implying that many users prefer shorter or partial charges rather than fully charging their vehicles. The long tail of the distribution reflects a minority of sessions requiring substantially greater energy.

**Fig. 3:**
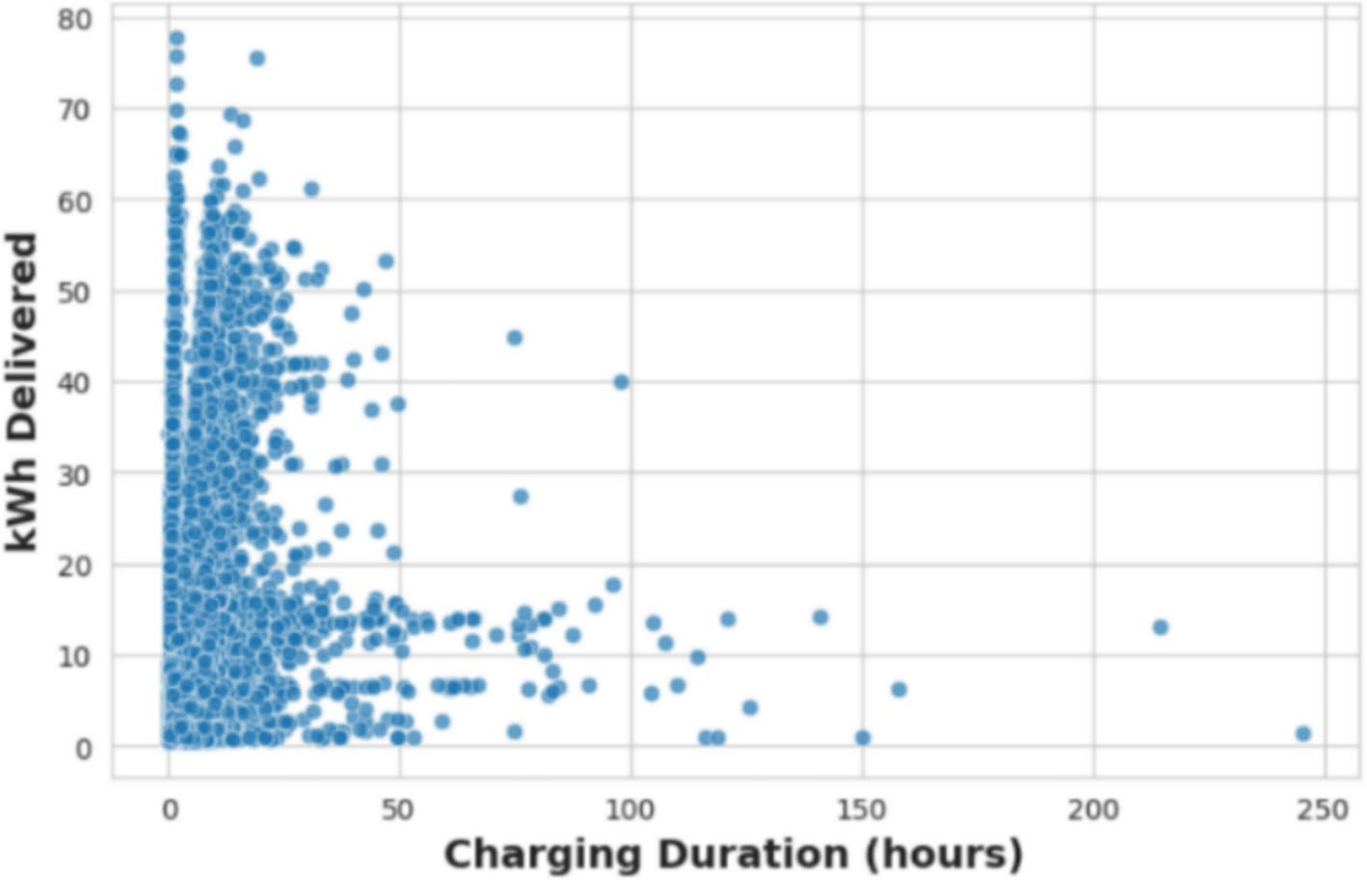
Charging duration vs. energy delivered.

Figure 3 illustrates the relationship between charging duration and the amount of energy dispensed. The majority of charging events are concentrated in the shorter duration range, between 0 and 50 h, with a broad spectrum of energy amounts delivered. The plot reveals a distinct right-skewed pattern, indicating the presence of outliers characterized by exceptionally long charging times coupled with minimal additional energy provided. This pattern suggests that while most users complete their charging relatively swiftly, a few engage in extended sessions. Such prolonged charging intervals with low energy output may point to irregularities or inefficiencies within the charging process.

**Fig. 4:**
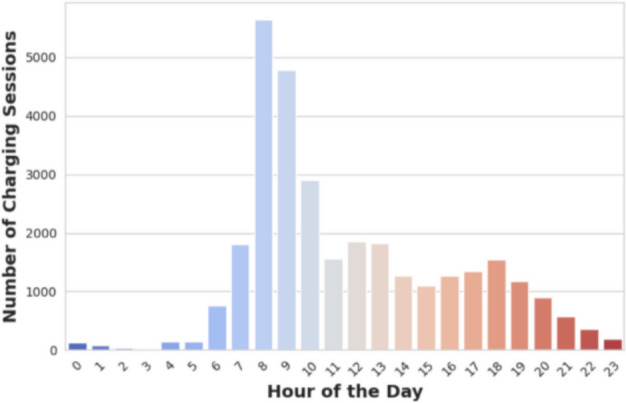
Daily charging sessions spread.

The distribution of charging sessions across different times of the day is depicted in Fig. 4. A pronounced surge in activity occurs between 8 and 10 AM, indicating that many users prefer to recharge their vehicles during the morning hours. After this peak, charging activity tends to stabilize through the afternoon and evening, gradually diminishing late at night and in the early morning. The notably lower charging frequencies during these off-peak hours likely reflect reduced demand or decreased vehicle usage during these periods.

**Fig. 5:**
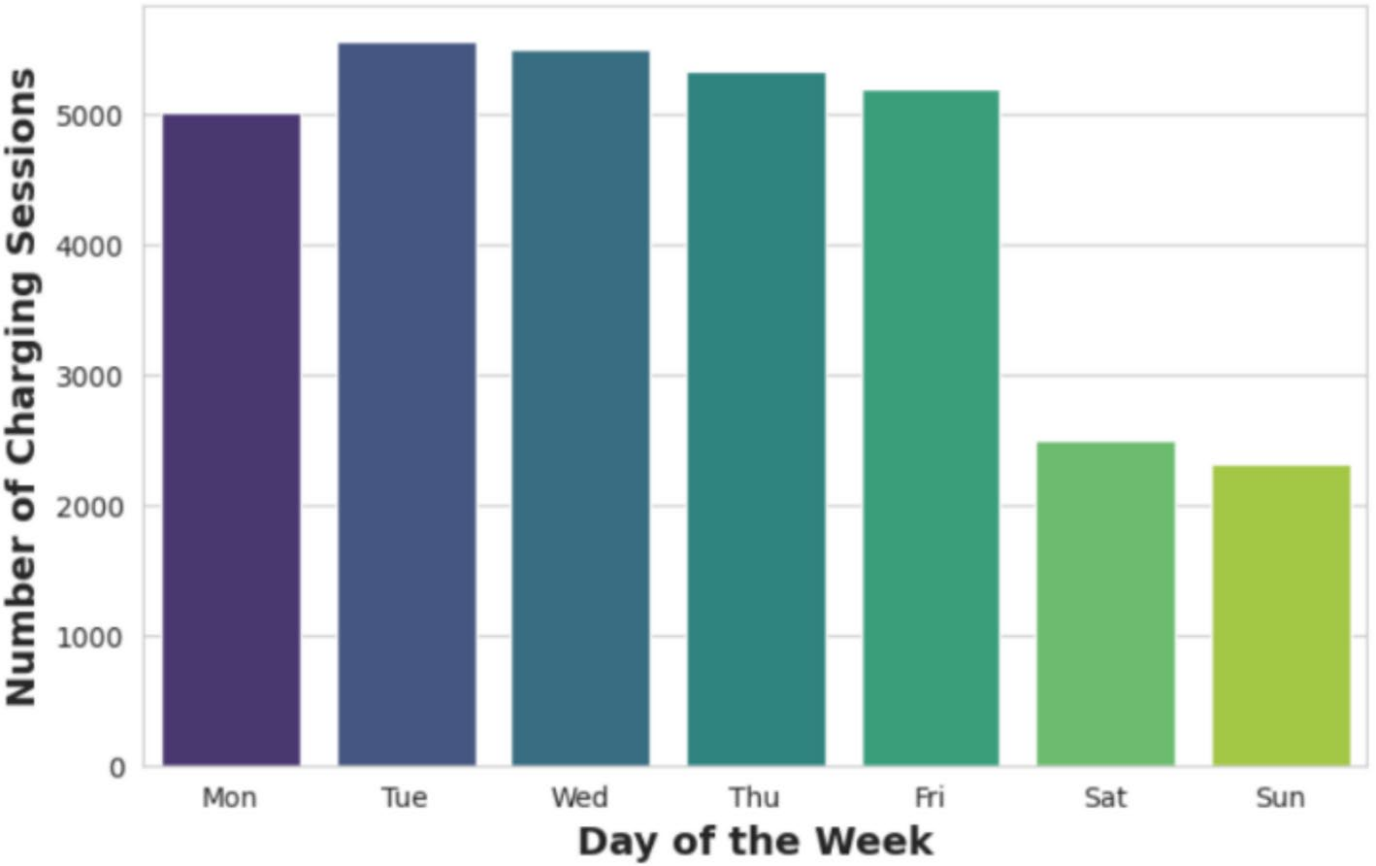
Charging sessions by day of the week.

**Fig. 6:**
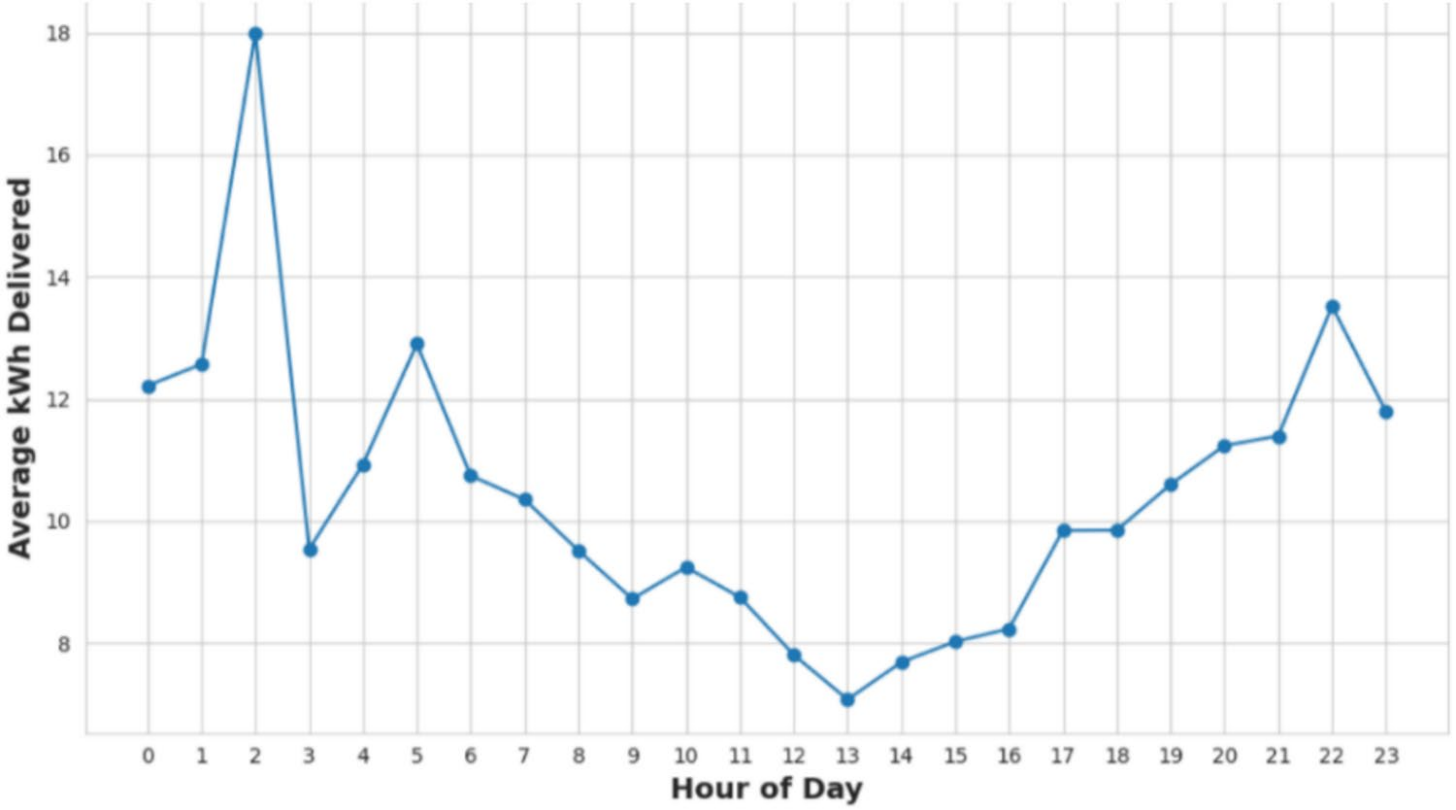
Average charging demand by hour of day.

Figure 5 displays the frequency of charging sessions by day of the week. The bar chart reveals higher charging activity throughout the workweek, Monday to Friday, with a peak occurring on Tuesday and Wednesday. In contrast, weekend days exhibit a significant decline in charging sessions. This pattern suggests that the typical workweek schedule strongly influences charging behavior, with users predominantly charging their vehicles during weekdays. Figure 6 illustrates the average charging demand by hour, based on the ACN dataset. The graph indicates a peak demand occurring at 2 AM, while the lowest demand is observed at 1 PM.

**Fig. 7:**
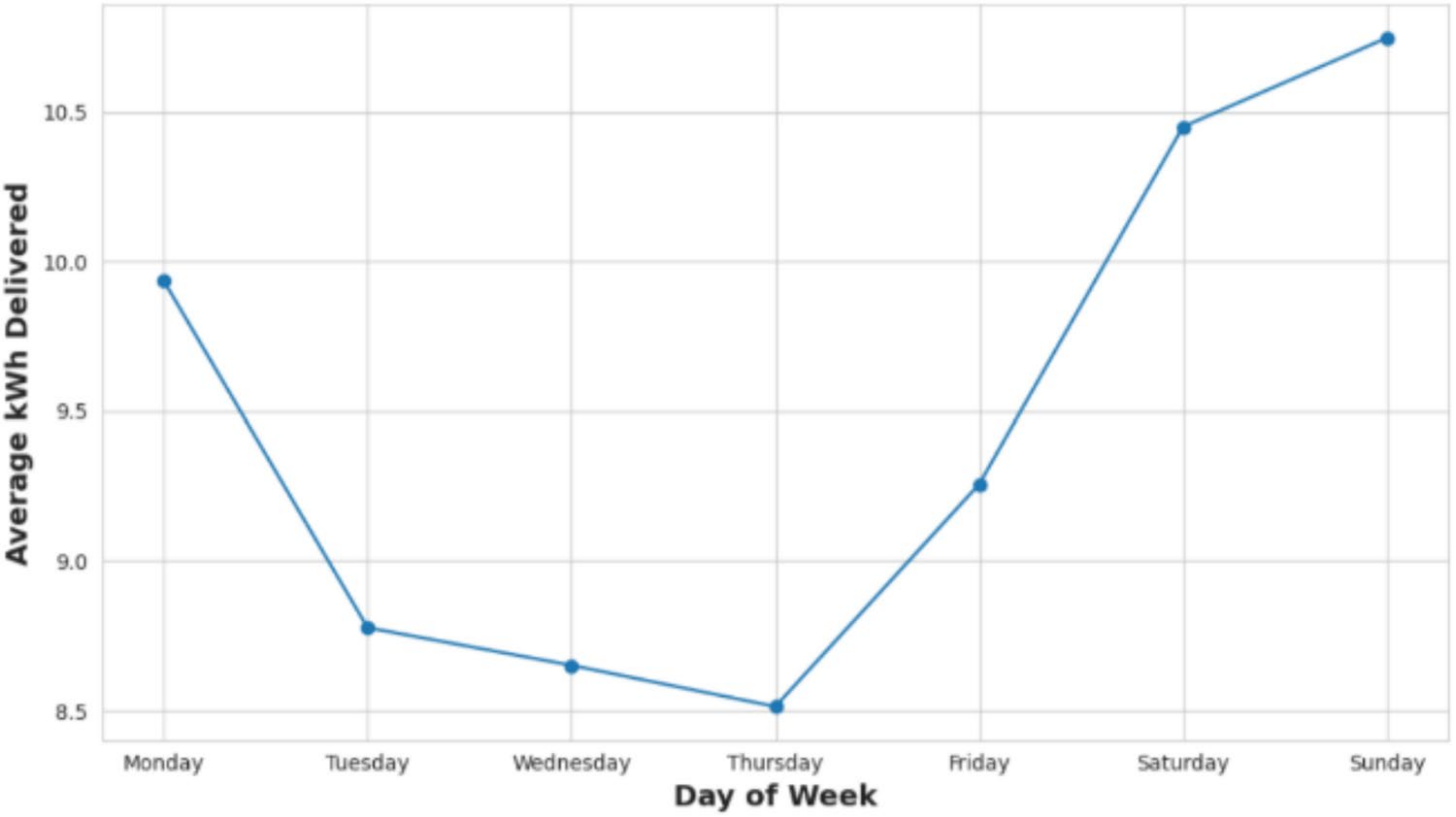
Average charging demand by day of week.

Figure 7 features a line graph portraying average daily charging demand across the week. The data shows a downward trend in demand from Monday through Thursday, reaching its lowest point on Thursday. Demand subsequently rises on Friday, culminating in the highest levels observed on Sunday. These patterns underscore the variability in charging behavior throughout the week and highlight the weekend as a period of increased energy needs. Such information is critical for effective load forecasting and the strategic placement of charging infrastructure to manage peak demand periods.

**Fig. 8:**
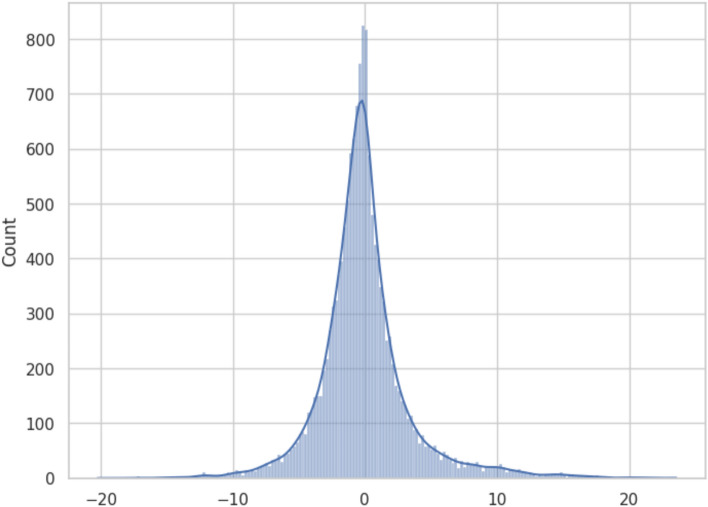
Residual distribution of the load forecasting model.

**Fig. 9:**
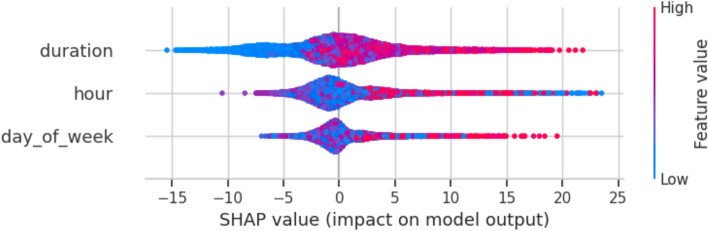
SHAP value.

Figure 8 visualizes the residuals, or prediction errors, of the load forecasting model. The residuals are predominantly centered around zero, indicating that the model’s predictions are largely unbiased. The distribution’s shape suggests that most errors are small; nonetheless, occasional larger deviations are evident, corresponding to under- or overestimations. Overall, the model’s residuals appear well-behaved, contributing to confidence in its predictive reliability. The SHAP summary plot (Fig. 9) provides a model-agnostic interpretation of feature impacts, corroborating the Gini-based importance rankings in Table 9. It confirms that charging_duration is the overwhelmingly dominant predictor of EV charging load, with longer sessions exhibiting a strong positive correlation with higher energy delivery. This primacy of session-level features over temporal ones like hour and day_of_week provides a crucial explanation for the model performance patterns observed throughout this study. The reliance on charging_duration elucidates why tree-based models like XGBoost and LightGBM performed robustly, as they excel at capturing such strong, non-linear, tabular relationships. Furthermore, it explains the high weight assigned to the xgb_pred component within the Hybrid 3 ensemble (Table 9), as the meta-learner optimally leverages XGBoost’s strength in modeling this key feature. This finding also offers a post-hoc interpretation of the ANOVA results (Table 12), which indicated no statistically significant difference in performance among the top models. The dominance of a single feature suggests that any model capable of effectively learning the charging_duration relationship will achieve competitive accuracy, thereby converging on similar performance levels and resulting in a non-significant ANOVA p-value. While this alignment with domain knowledge—where longer sessions logically transfer more energy—validates the model’s logic, it also highlights a potential limitation. The model’s performance is heavily contingent on accurate duration data, which may not always be available for long-horizon forecasts. Furthermore, the overshadowing of temporal features indicates that the current models may be less effective at predicting load variations driven purely by time-of-use patterns or external factors like grid events, pointing to a area for future work where incorporating additional contextual variables could yield improvements.

### Preprocessing

All models were trained with a fixed random seed (seed = 42) to ensure full reproducibility. The ACN–Caltech site dataset was randomly split into 80% for training and 20% for testing without shuffling, maintaining temporal consistency and representative sampling. The electric vehicle (EV) charging session dataset from the ACNPortal was preprocessed to enhance its suitability for predicting energy consumption (kWhDelivered), focusing on data cleaning, feature engineering, outlier management, and missing value handling to support robust model performance. Initial cleaning removed irrelevant identifier columns (_id, sessionID, stationID, spaceID, siteID, clusterID, userID) to streamline analysis, while datetime columns (connectionTime, disconnectTime, doneChargingTime) were converted to a timezone-naive format to ensure consistent temporal processing. Feature engineering enriched the dataset with temporal and derived variables: hour (0–23), day_of_week (0–6), and month (1–12) were extracted from connectionTime; a binary is_weekend feature (1 for Saturday/Sunday, 0 otherwise) and a season feature (0–3, mapping months to Winter, Spring, Summer, Fall) were created to capture weekly and seasonal patterns. Cyclical features (hour_sin, hour_cos) modeled the 24-h cycle, while day_of_year (1–365) and week_of_year (1–52) represented annual and weekly trends. A binary is_holiday feature (1 for December/January, 0 otherwise) accounted for holiday effects. Charging-related features included duration (disconnectTime minus connectionTime, in hours) and charging_duration (doneChargingTime minus connectionTime, in hours), with charging_duration_log applied to mitigate skewness, as confirmed by a skewness value of 1.24 (Table 2). Interaction terms (hour_charging_interaction, weekend_charging_interaction) and lagged features (lag_1_log, lag_2_log, lag_3_log) based on log-transformed duration, along with rolling_mean_3_log and rolling_mean_5_log, captured historical patterns, with non-numeric values coerced to NaN and converted to float64.

**Table 2:** Feature skewness analysis.

Feature	Skewness value
hour	0.87
day_of_week	0.31
month	− 0.04
season	0.00
duration	0.60
charging_duration	1.24
charging_duration_log	0.70
hour_sin	− 0.66
hour_cos	1.47
day_of_year	− 0.03
week_of_year	− 0.02
is_holiday	1.46
lag_1_log	− 0.18
lag_2_log	− 0.19
lag_3_log	− 0.19
rolling_mean_3_log	− 0.33
rolling_mean_5_log	− 0.38
kWhDelivered (Target)	1.09

Skewness measures for all features and the target variable. Values > 1 or < -1 indicate significant skewness requiring transformation.

Outlier management employed the Interquartile Range (IQR) method, calculating Q1 and Q3 for duration, charging_duration, and kWhDelivered, defining the IQR as Q3 minus Q1, and capping values between Q1—1.5 × IQR and Q3 + 1.5 × IQR to reduce the impact of extreme values. A copy of the original dataset (df_original) was preserved for comparative analysis. Missing values, arising from feature engineering or lags, were removed, and the original dataset was aligned to the cleaned dataset’s indices. Table 2 (Feature Skewness Analysis) guided transformations, revealing significant skewness (> 1 or < -1) for charging_duration (1.24), hour_cos (1.47), and is_holiday (1.46), and moderate skewness for kWhDelivered (1.09), prompting a log transform for the target to improve model fit. A data leakage check confirmed no identical rows between training and test sets by converting data to tuples and checking set intersections, preserving temporal integrity. The preprocessed dataset, comprising 17 features (hour, day_of_week, month, season, duration, charging_duration, charging_duration_log, hour_sin, hour_cos, day_of_year, week_of_year, is_holiday, lag_1_log, lag_2_log, lag_3_log, rolling_mean_3_log, rolling_mean_5_log) and kWhDelivered as the target, was thus optimized for training and evaluating machine learning, deep learning, and ensemble models.

### Models

#### XGBoost (eXtreme Gradient Boosting)

The XGBoost algorithm, introduced by Tianqi Chen in 2016, is a powerful decision tree–based ensemble method that utilizes the boosting technique to improve predictive performance^28^. The XGBoost algorithm addresses these challenges by incorporating enhancements including tree pruning, parallel processing, and the introduction of regularization terms, thereby improving the overall robustness and efficiency of the model.

#### BiLSTM

**Fig. 10:**
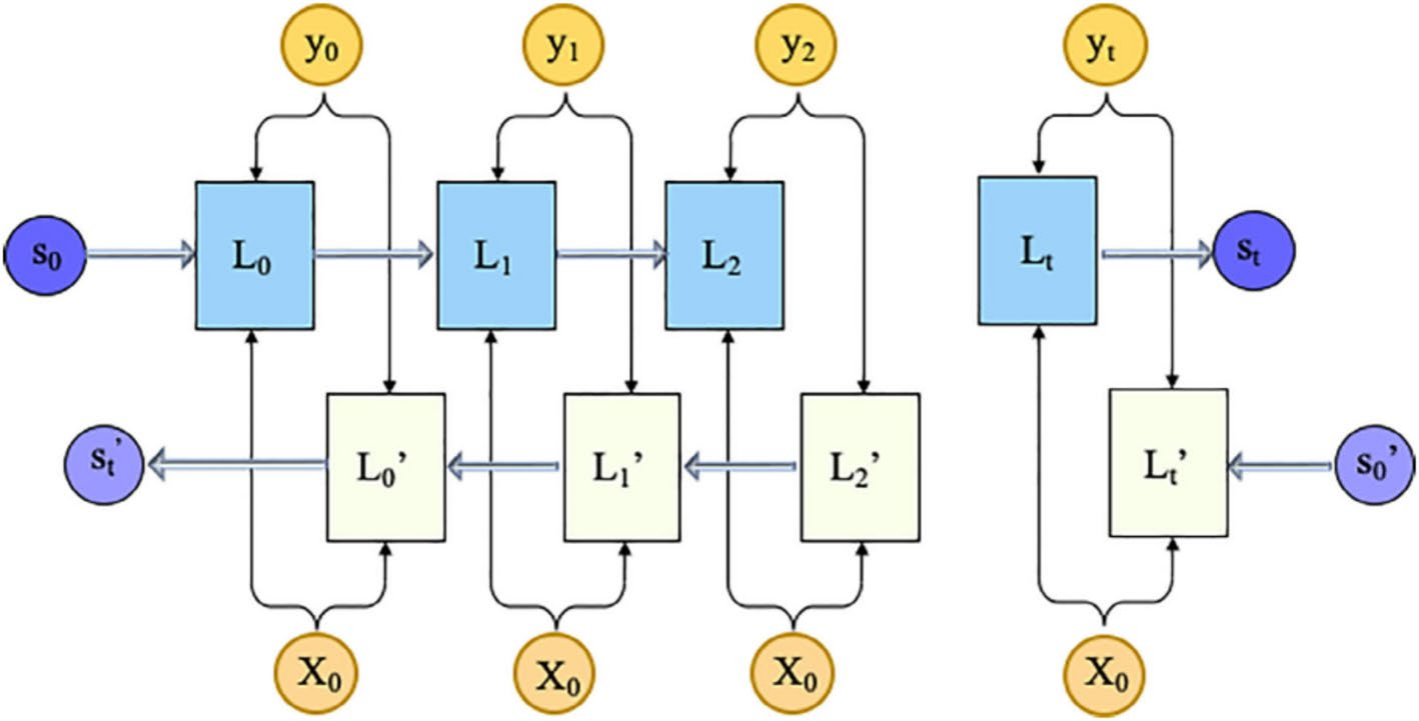
Schematic diagram of BiLSTM.

BiLSTM networks comprise two distinct LSTM layers arranged to process sequential data in opposite temporal directions. Specifically, one layer analyzes the input sequence in the forward direction, while the other simultaneously traverses it in reverse, thereby enabling the model to capture contextual information from both past and future states within the sequence^29^. These two outputs are then combined to produce the final prediction. Unlike a standard LSTM, which analyzes time-dependent data in only one direction, BiLSTM incorporates this additional reverse pass to capture patterns and dependencies that may be overlooked when considering the sequence in a single direction. The architecture of the BiLSTM approach is shown in Fig. 10, where *L*_*i*_ denotes the forward LSTM layer, *L*_*i*_ represents the backward LSTM layer, and *s* and *s*′ correspond to the time series information propagated through the respective LSTM cells.

#### XGBoost and Bi-LSTM (Hybrid 3 model)

The Hybrid 3 model combines the strengths of XGBoost and Bidirectional Long Short-Term Memory (BiLSTM) networks to enhance the prediction of energy consumption (kWhDelivered) in the electric vehicle (EV) charging session dataset. BiLSTM excels at capturing temporal dependencies and sequential patterns in time-series data, leveraging its bidirectional architecture to model relationships in both forward and backward directions across the 17 preprocessed features (e.g., hour, charging_duration_log, lag_1_log). Conversely, XGBoost is adept at handling structured data, capturing complex nonlinear interactions among features through gradient boosting and tree-based learning. By integrating these models, Hybrid 3 aims to combine BiLSTM’s sequential modeling capabilities with XGBoost’s robust feature interaction modeling to achieve superior predictive accuracy compared to standalone models.

The Hybrid 3 model was implemented as a stacking ensemble. First, predictions were generated independently from two base models: (1) an optimized XGBoost model, tuned using Optuna with parameters such as n_estimators, max_depth, and learning_rate trained on the scaled feature set (X_train_scaled); and (2) a BiLSTM model with three bidirectional LSTM layers (128, 64, and 64 units, respectively), regularized with L2 regularization (0.0001) and dropout (0.07), trained on the 3D input (X_train_lstm) reshaped to include a time-step dimension. These predictions (xgb_pred_train, bilstm_pred_train for training; xgb_pred_test, bilstm_pred_test for testing) were stacked column-wise to form a meta-feature matrix (X_meta_train, X_meta_test). A meta-learner, implemented as an XGBoost model with default parameters and a random seed of 42, was trained on X_meta_train to predict the target (y_train), effectively learning to weigh the contributions of the base models’ predictions. The meta-learner then generated final predictions on X_meta_test for evaluation.

This stacking approach leverages BiLSTM’s ability to model temporal dynamics, such as diurnal and seasonal charging patterns, while incorporating XGBoost’s strength in capturing feature interactions, such as those between hour_charging_interaction and charging_duration_log. The model was trained on the preprocessed dataset and evaluated using metrics including Mean Absolute Error (MAE), Mean Squared Error (MSE), Root Mean Squared Error (RMSE), and R^2^, with performance assessed on both cleaned and original (pre-outlier-capping) datasets to ensure robustness The training and testing times were recorded as hybrid 3_training_time and hybrid 3_testing_time, respectively, to assess computational efficiency. This hybrid framework was designed to mitigate the limitations of individual models, delivering more reliable and precise predictions for EV charging energy demand forecasting.

**Fig. 11:**
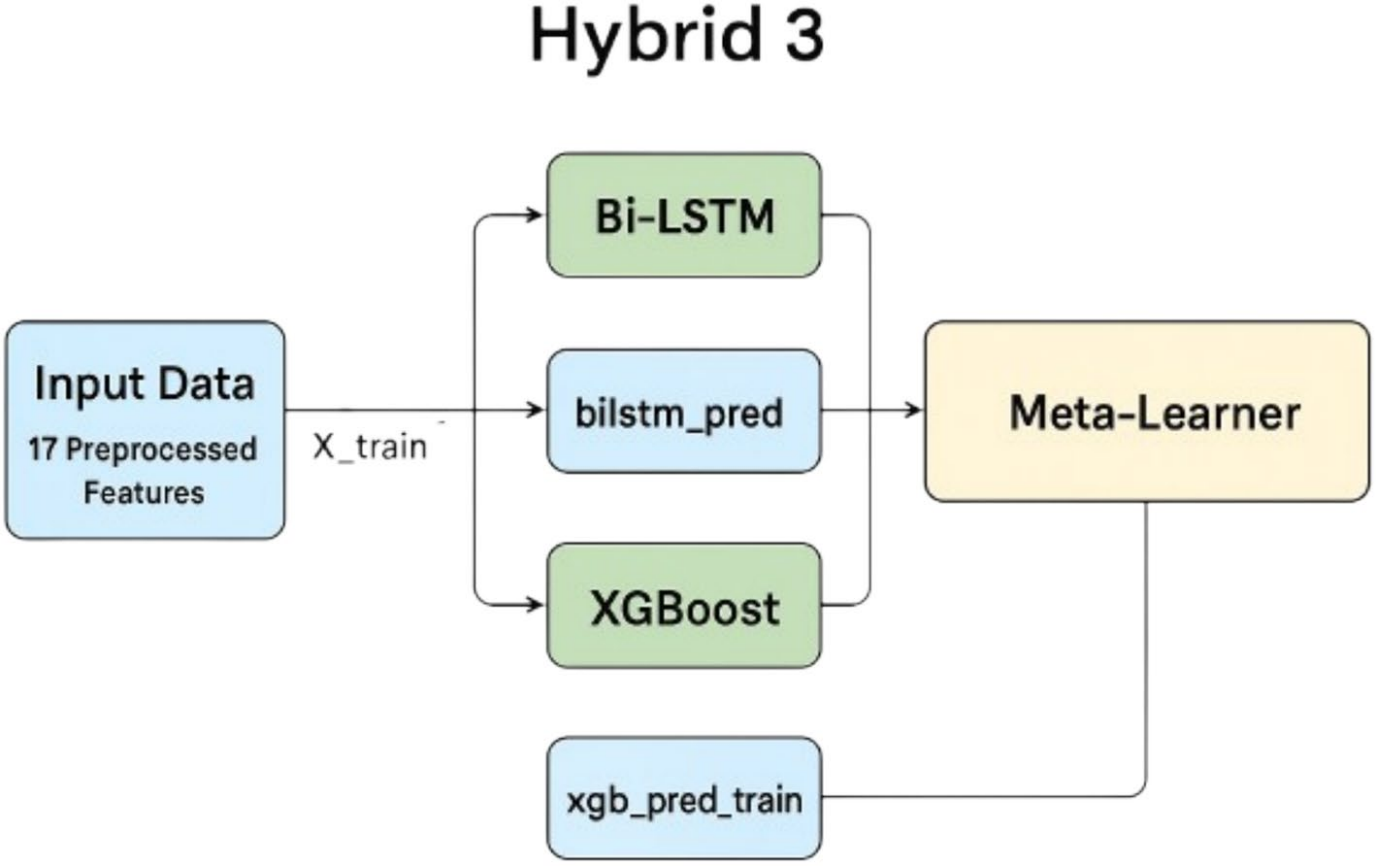
Architecture of the hybrid 3 (XGBoost–BiLSTM) stacking ensemble model.

Figure 11 illustrates the architecture of the proposed Hybrid 3 model, which integrates an optimized XGBoost regressor and a BiLSTM neural network within a stacking ensemble framework for short-term EV charging load forecasting.

In the first stage, the preprocessed feature set is independently input into two base learners:

- XGBoost, which captures nonlinear relationships and feature interactions, and
- BiLSTM, which models temporal dependencies and sequential patterns across charging sessions.

Each base model generates preliminary predictions that are concatenated to form a meta-feature matrix. This matrix is then used to train an XGBoost meta-learner, which learns optimal weights for combining the base models’ outputs to generate the final prediction.

This hybrid design leverages BiLSTM’s ability to model time-series dynamics alongside XGBoost’s strength in structured feature learning, thereby improving both accuracy and robustness in EV energy demand forecasting.

#### Other baseline models

This study evaluates 24 baseline models spanning statistical, machine learning, deep learning, and ensemble categories to provide comprehensive benchmarking against the proposed Hybrid 3 model. All baseline models were implemented with architectures and hyperparameters specified in Table 3, ensuring fair comparison across diverse methodologies.

**Table 3:** Hyperparameter search ranges and fixed values.

Model	Hyperparameter	Range/Value	Notes
XGBoost	n_estimators	1000–3000 (step 200)	Number of boosting rounds
	max_depth	4–8	Maximum tree depth
	learning_rate	0.005–0.1 (log scale)	Step size shrinkage
	subsample	0.7–0.95	Subsample ratio of the training instances
	colsample_bytree	0.7–0.95	Subsample ratio of columns per tree
	colsample_bylevel	0.7–0.95	Subsample ratio of columns per level
	gamma	0–0.3	Minimum loss reduction for split
	min_child_weight	1–10	Minimum sum of instance weight
	reg_alpha	0–5	L1 regularization term
	reg_lambda	0.5–5	L2 regularization term
LightGBM	n_estimators	10–100 (step 10)	Number of boosting iterations
	max_depth	2–5	Maximum tree depth
	learning_rate	0.1–0.3 (log scale)	Step size shrinkage
	num_leaves	10–50	Maximum number of leaves per tree
	subsample	0.5–0.9	Subsample ratio of the training instances
	colsample_bytree	0.5–0.9	Subsample ratio of columns per tree
	min_child_samples	20–100	Minimum number of data in a leaf
	reg_alpha	0–5	L1 regularization term
	reg_lambda	0.5–5	L2 regularization term
	subsample_freq	1–10	Frequency of subsample
BiLSTM	units	128, 64, 64	Units per LSTM layer (fixed architecture)
	num_layers	3	Number of LSTM layers (fixed)
	learning_rate	0.0005	Fixed learning rate for AdamW
	batch_size	128	Fixed batch size
	dropout	0.07	Dropout rate (fixed)
	l2_regularization	0.0001	L2 regularization (fixed)
CNN	filters	64, 32	Filters per convolutional layer (fixed)
	kernel_size	3	Kernel size (fixed)
	learning_rate	0.001	Fixed learning rate for AdamW
	batch_size	128	Fixed batch size
TCN	nb_filters	64	Number of filters (fixed)
	kernel_size	3	Kernel size (fixed)
	dilations	[1, 2, 4]	Dilation rates (fixed)
	learning_rate	0.001	Fixed learning rate for AdamW
	batch_size	128	Fixed batch size
Transformer	num_heads	4	Number of attention heads (fixed)
	key_dim	64	Dimension of attention keys (fixed)
	num_layers	2	Number of attention layers (fixed)
	learning_rate	0.001	Fixed learning rate for AdamW
	batch_size	128	Fixed batch size
SARIMAX	p (AR order)	1	Autoregressive order (fixed)
	d (differencing)	1	Differencing order (fixed)
	q (MA order)	1	Moving average order (fixed)
	seasonal_order	(1, 1, 1, 24)	Seasonal order (fixed, 24-h period)
Prophet	yearly_seasonality	True	Fixed setting
	weekly_seasonality	True	Fixed setting
	daily_seasonality	True	Fixed setting

Hyperparameter ranges for XGBoost and LightGBM were tuned using Optuna with TimeSeriesSplit (3 folds for XGBoost, variable for LightGBM). Neural network models (BiLSTM, CNN, TCN, Transformer) use fixed architectures with specified hyperparameters, optimized via early stopping and learning rate reduction. SARIMAX and Prophet use fixed configurations.

Statistical Baselines: Four traditional time series models were implemented as performance baselines. The Persistence model uses the previous observation as the forecast (y_t = y_{t-1}), serving as a naive benchmark. Seasonal Naïve applies 24-h periodicity forecasting (y_t = y_{t-24}), capturing diurnal patterns. SARIMAX employs an autoregressive integrated moving average model with exogenous variables using order (1,1,1) and seasonal order (1,1,1,24) to account for hourly patterns. Prophet implements additive time series modeling with configurable yearly, weekly, and daily seasonality components, incorporating all 17 engineered features as regressors.

Deep Learning Baselines: Three advanced neural architectures complement the BiLSTM model. The Convolutional Neural Network (CNN) uses two convolutional layers (64 and 32 filters, kernel size 3) followed by max pooling and dense layers, capturing local temporal patterns through 1D convolutions. The Temporal Convolutional Network (TCN) implements dilated convolutions with 64 filters, kernel size 3, and dilation rates^1,2,4^ to model long-range dependencies efficiently. The Transformer model employs two multi-head attention layers (4 heads, key dimension 64) with layer normalization and residual connections, leveraging self-attention mechanisms to capture global temporal dependencies across the input sequence.

Machine Learning Baselines: Two gradient boosting frameworks provide robust nonlinear modeling. XGBoost (eXtreme Gradient Boosting) uses tree-based ensemble learning with hyperparameters optimized via Optuna (Table 4), incorporating early stopping and L1/L2 regularization for robustness. LightGBM (Light Gradient Boosting Machine) employs histogram-based learning with leaf-wise tree growth, also tuned via Optuna, offering superior computational efficiency through gradient-based one-side sampling.

**Table 4:** Optimal hyperparameters for XGBoost and LightGBM.

Parameter	XGBoost	LightGBM
n_estimators	1400	100
max_depth	6	4
learning_rate	0.007297	0.131602
subsample	0.810629	0.767120
colsample_bytree	0.905182	0.832741
colsample_bylevel	0.749766	–
gamma	0.177765	–
min_child_weight	6	–
min_child_samples	–	38
reg_alpha	0.525283	4.389436
reg_lambda	3.978445	1.668866
subsample_freq	–	9

Ensemble Baselines: Twelve ensemble methods systematically combine base learners to explore integration strategies. Boosting ensembles implement sequential residual learning, where each model corrects the predecessor’s errors through weighted averaging (equal weights). Bagging ensembles use bootstrap aggregation with 10 estimators trained in parallel, reducing variance through averaging. Stacking ensembles employ meta-learning architectures where base model predictions serve as features for a final XGBoost meta-learner. Weighted combinations apply optimized linear combinations with fixed weights (0.6/0.4 for two models, 0.4/0.3/0.3 for three models), representing both weighted sum and blending approaches.

Alternative Hybrid Baselines: Two additional hybrid architectures provide comparative analysis of XGBoost-BiLSTM integration strategies. Hybrid 1 implements feature concatenation, where XGBoost’s tree structure outputs are stacked with original features to form an expanded input for the BiLSTM network, creating a 18-dimensional feature space. Hybrid 2 uses sequential prediction fusion, extracting latent representations from BiLSTM’s intermediate layers (excluding final dense layers) as sequential features for subsequent XGBoost regression, effectively transferring temporal knowledge through dimensionality-reduced embeddings.

All baseline models were evaluated using the comprehensive framework outlined in Sect. “Evaluation metrics”, with performance metrics computed on both cleaned and original datasets to assess robustness. Hyperparameter optimization employed TimeSeriesSplit cross-validation (3 folds for tree-based models), early stopping for neural networks, and fixed configurations for statistical models, ensuring methodological consistency across the 25-model comparison.

The following Table 3 outlines the hyperparameter ranges or fixed values used for tuning and training the models in this study, optimized for forecasting EV charging load (kWhDelivered) using the ACN dataset.

Table 4 below lists the best hyperparameters for the XGBoost and LightGBM models, obtained through Optuna optimization with TimeSeriesSplit.

### External validation dataset

This dataset comprises 1,965,239 simulated charging sessions representing a distinct, generic EVCS site, designed to assess domain-shift effects in a controlled yet realistic setting.

The dataset was preprocessed to align with the ACN schema. Energy delivery values (kWhDelivered) were clipped to a realistic EV range (0–100 kWh) to remove synthetic outliers, and temporal features (hour, day_of_week, month, season, is_holiday, is_weekend) were reconstructed from connection timestamps. Missing environmental context was addressed by generating synthetic meteorological variables—temperature (T ~ N(20 C, 5)) and humidity (H ~ N(60%, 15))—using seeded Gaussian noise (seed = 42) for reproducibility. A synthetic timeline was constructed by combining day_of_week and connectionTime_decimal to form a continuous chronological sequence spanning multiple simulated years.

Descriptive statistics of the cross-site dataset prior to preprocessing are summarized in Table 5. The distribution of kWhDelivered exhibits a right-skewed profile typical of partial EV charges (mean = 9.44 kWh, SD = 5.70), while session durations vary widely (mean = 3.80 h). These characteristics confirm that the dataset adequately reflects heterogeneous charging behaviors necessary for robust cross-site evaluation. Results from this external validation are reported in Sect. “External validation results”.

**Table 5:** Descriptive statistics of the cross-site dataset (pre-processing).

Statistic	connectionTime_decimal	charging_duration	kWhDelivered	day_of_week
Count	1,965,239	1,965,239	1,965,239	1,965,239
Mean	14.49	3.80	9.44	14,800.03
Std	6.43	3.39	5.70	8,531.91
Min	0.00	0.00	0.00	1.00
25%	14.25	1.44	4.87	7,424.00
50%	16.17	2.55	9.17	14,793.00
75%	18.04	5.44	13.57	22,198.00
Max	24.00	39.41	62.54	29,600.00

### Computational environment

All computational experiments were performed on a system utilizing two NVIDIA Tesla T4 GPUs (16 GB VRAM each) with CUDA Version 12.6 and an x86_64 CPU. Deep learning models (BiLSTM, CNN, Transformer) were accelerated using TensorFlow and with GPU support, while ensemble methods (XGBoost, LightGBM) primarily leveraged CPU resources.

## Evaluation metrics

To evaluate the performance of the proposed models for forecasting energy delivered (kWhDelivered) in EV charging sessions, we employed a suite of standard regression metrics: Mean Absolute Error (MAE), Mean Squared Error (MSE), Root Mean Squared Error (RMSE), and the Coefficient of Determination (R^2^). These metrics were calculated for both training and testing datasets, with evaluations conducted on both the cleaned dataset (after outlier removal and preprocessing) and the original dataset (before outlier removal) to assess model robustness. Additionally, walk-forward validation and statistical testing were applied to ensure robust and reliable performance comparisons across models. Below, we describe each metric, its mathematical formulation, its relevance to the task, and the evaluation methodology.

### Mean squared error (MSE)

The MSE quantifies the average of the squared deviations between the observed and predicted values. A lower MSE indicates superior predictive accuracy, with an ideal value of zero representing a perfect correspondence between the actual and forecasted data.

1 $$MSE=\frac{1}{n}\sum_{i=1}^{n}{\left({y}_{i}-{\widehat{y}}_{i}\right)}^{2}$$

where, *n* denotes the total number of observations considered, ${\widehat{{\varvec{y}}}}_{{\varvec{i}}}$ represents the predicted or forecasted value, and ${{\varvec{y}}}_{{\varvec{i}}}$ corresponds to the true or observed value.

### Root mean squared error (RMSE)

The RMSE is defined as the square root of the MSE and possesses the advantage of being expressed in the same units as the target variable, thereby facilitating interpretability.

2 $$RMSE=\sqrt{\frac{1}{n}\sum_{i=1}^{n}{\left({y}_{i}-{\widehat{y}}_{i}\right)}^{2}}$$

### Mean absolute error (MAE)

This metric represents the average of the absolute deviations between the observed values and their corresponding forecasts, providing a direct measure of prediction accuracy in terms of magnitude of errors.

3 $$MAE=\frac{1}{n}\sum_{i=1}^{n}\left|{y}_{i}-{\widehat{y}}_{i}\right|$$

### Coefficient of determination (R^2^)

The coefficient of determination, commonly referred to as R-squared (R^2^), evaluates the goodness-of-fit of a predictive model by quantifying the proportion of variance in the observed data that is explained by the model’s predictions. This metric ranges between 0 and 1, where a value of 0 indicates that the model fails to capture the underlying data patterns, reflecting poor fit, and a value of 1 denotes perfect prediction accuracy with no deviation from the actual observations.

4 $${R}^{2}=1-\frac{\sum_{i=1}^{M}{\left({\widehat{y}}_{i}-{y}_{i}\right)}^{2}}{\sum_{1=1}^{M}{\left(\overline{{y }_{i}}-{y}_{i}\right)}^{2}}$$

### Walk-forward validation

To further validate model performance in a time-series context, walk-forward validation was conducted using a fivefold TimeSeriesSplit. This approach simulates rolling forecasts by training on progressively larger subsets of the data and testing on subsequent time periods, ensuring temporal consistency. The top-performing models (XGBoost, LightGBM, BiLSTM, Stacking (XGBoost, BiLSTM, LightGBM), and Hybrid 3 (XGBoost, BiLSTM)) were evaluated, with metrics (MAE, MSE, RMSE, R^2^) calculated for each fold. The mean and standard deviation of these metrics across folds were reported to assess model stability and generalization. Walk-forward validation is particularly relevant for EV charging forecasting, as it reflects the dynamic nature of charging session data over time.

### Statistical testing

To determine whether performance differences between models were statistically significant, a one-way ANOVA test was performed on the MAE values from walk-forward validation. The ANOVA test evaluates the null hypothesis that all models have the same mean MAE, with a p-value threshold of 0.05. The effect size (${\upeta }^{2}$) was calculated to quantify the magnitude of differences:

5 $${\upeta }^{2}=\frac{F\cdot (k-1)}{F\cdot \left(k-1\right)+n}$$

where $F$ is the ANOVA F-statistic, $k$ is the number of models, and $n$ is the total number of observations. An ${\upeta }^{2}$ value below 0.06 indicates a small effect, 0.06–0.14 a medium effect, and above 0.14 a large effect. Additionally, 95% confidence intervals for MAE were computed for each model using the t-distribution to assess the precision of performance estimates. These statistical analyses ensure robust comparisons and guide model selection.

### Computational efficiency

In addition to predictive performance, computational efficiency was evaluated by measuring tuning time, training time, and testing time for each model. These metrics, recorded in seconds, are critical for practical deployment in real-time EV charging systems, where low latency is essential.

The computational efficiency of the models, as detailed in Table 6, is a critical factor for their practical deployment in real-time EV charging systems, where low latency is essential for dynamic load management. Training and testing times, measured in seconds, were recorded for both cleaned and original datasets, with tuning times reported for XGBoost and LightGBM via Optuna. The results reveal significant variability across models, reflecting differences in algorithmic complexity and ensemble strategies.

**Table 6:** Training and testing times of models.

Model	Training time (s)	Testing time (s)
SARIMAX	392.72	0.30
Prophet	3.12	2.27
BiLSTM	103.78	0.47
CNN	21.17	0.42
TCN	24.82	0.59
Transformer	44.29	0.90
XGBoost	1.90	0.03
LightGBM	0.10	0.01
Boosting (XGBoost, BiLSTM)	2.29	0.00
Bagging (XGBoost, BiLSTM)	21.41	1.81
Stacking (XGBoost, BiLSTM)	517.29	3.71
Boosting (XGBoost, BiLSTM, LightGBM)	0.04	0.00
Bagging (XGBoost, BiLSTM, LightGBM)	1.02	0.36
Stacking (XGBoost, BiLSTM, LightGBM)	531.56	3.85
Bagging (LightGBM, BiLSTM)	1.04	0.41
Stacking (LightGBM, BiLSTM)	536.24	4.20
Hybrid 1 (XGBoost, BiLSTM)	1471.21	46.70
Hybrid 2 (XGBoost, BiLSTM)	6.77	0.18
Hybrid 3 (XGBoost, BiLSTM)	2.52	0.01

LightGBM stands out as the most computationally efficient model, with a training time of 0.10 s and a testing time of 0.01 s, making it highly suitable for scenarios requiring rapid updates. XGBoost follows with a training time of 1.90 s and a testing time of 0.03 s, benefiting from its optimized tree-boosting implementation. Among hybrid and ensemble models, Hybrid 3 (XGBoost, BiLSTM) achieves a commendable balance, with a training time of 2.52 s and a testing time of 0.01 s, closely aligning with the efficiency of standalone XGBoost. This efficiency is attributed to the stacking ensemble’s use of precomputed base model predictions, reducing redundant computations during testing.

In contrast, more complex ensemble models exhibit higher computational demands. Stacking (XGBoost, BiLSTM, LightGBM) requires 531.56 s for training and 3.85 s for testing, reflecting the overhead of training multiple base models and a meta-learner. Hybrid 1 (XGBoost, BiLSTM) is the least efficient, with a training time of 1471.21 s and a testing time of 46.70 s, likely due to its unique implementation or resource-intensive optimization. Statistical models like SARIMAX (392.72 s training, 0.30 s testing) and deep learning models like BiLSTM (103.78 s training, 0.47 s testing) also show higher training times, though their testing times remain low, suitable for inference.

The low testing times across most models (e.g., 0.00–0.90 s) suggest feasibility for real-time applications, with Hybrid 3’s 0.01-s testing time indicating potential for near-instantaneous predictions. However, training times for complex models (e.g., Stacking, Hybrid 1) highlight the need for offline or periodic retraining rather than on-the-fly updates. The efficiency of Hybrid 3, combined with its competitive predictive performance (MAE of 2.6870 kWh on cleaned data), positions it as a practical choice for EV charging load forecasting, especially when balanced against accuracy requirements. Future work could explore parallelization or lightweight variants to further reduce training times for scalable deployment.

## Results and discussion

This section presents a comprehensive evaluation of the models developed for forecasting energy delivered (kWhDelivered) at electric vehicle charging stations (EVCS), utilizing the Adaptive Charging Network (ACN) dataset. The analysis compares model performance on cleaned and original datasets using Mean Absolute Error (MAE), Mean Squared Error (MSE), Root Mean Squared Error (RMSE), and the Coefficient of Determination (R^2^). Subsequent subsections assess temporal robustness through walk-forward validation, statistical significance via ANOVA, and computational efficiency for practical deployment. The discussion concludes with implications, recommendations, and limitations, drawing on data from Tables 7, 8, 9, 10, 11, 12, 13 and supplementary analyses.

**Table 7:** Models performance on cleaned data.

Model	Split	MAE (kWh)	MSE (kW $${h}^{2})$$	RMSE (kWh)	R^2^
Persistence	Train	7.6588	99.9651	9.9983	− 0.8929
	Test	6.7029	85.9118	9.2689	− 0.9525
Seasonal Naïve	Train	7.7371	101.8763	10.0934	− 0.9291
	Test	6.7364	88.0915	9.3857	− 1.0021
SARIMAX	Train	4.9714	3319.7214	57.6170	− 62.0726
	Test	2.8947	17.4591	4.1784	0.6032
Prophet	Train	4.7899	3038.2325	55.1202	− 56.5301
	Test	2.8441	16.4564	4.0566	0.6260
BiLSTM	Train	3.0548	19.9111	4.4622	0.6230
	Test	2.7816	17.8198	4.2213	0.5950
CNN	Train	3.1823	20.3922	4.5158	0.6139
	Test	2.6859	16.1456	4.0182	0.6331
TCN	Train	2.9141	17.9306	4.2345	0.6605
	Test	2.9802	19.7211	4.4408	0.5518
Transformer	Train	5.6594	59.8620	7.7371	− 0.1335
	Test	4.8141	44.2992	6.6558	− 0.0068
XGBoost	Train	2.9353	17.9511	4.2369	0.6601
	Test	2.6697	15.5640	3.9451	0.6463
LightGBM	Train	3.1619	20.2459	4.4995	0.6166
	Test	2.6523	15.1386	3.8908	0.6559
Boosting (XGBoost, BiLSTM)	Train	2.9329	18.2956	4.2773	0.6536
	Test	2.6646	16.1864	4.0232	0.6321
Bagging (XGBoost, BiLSTM)	Train	2.8386	16.6838	4.0846	0.6841
	Test	2.6845	15.8389	3.9798	0.6400
Stacking (XGBoost, BiLSTM)	Train	3.2254	22.9762	4.7933	0.5649
	Test	2.7749	18.1566	4.2611	0.5874
Weighted (XGBoost, BiLSTM)	Train	2.9249	18.1283	4.2577	0.6567
	Test	2.6558	15.9790	3.9974	0.6368
Boosting (XGBoost, BiLSTM, LightGBM)	Train	2.9919	18.7149	4.3261	0.6456
	Test	2.6432	15.6764	3.9593	0.6437
Bagging (XGBoost, BiLSTM, LightGBM)	Train	3.1650	20.1545	4.4894	0.6184
	Test	2.6631	15.4393	3.9293	0.6491
Stacking (XGBoost, BiLSTM, LightGBM)	Train	3.0937	21.1595	4.6000	0.5993
	Test	2.7776	17.6877	4.2057	0.5980
Weighted XGBoost, BiLSTM, LightGBM)	Train	2.9843	18.6087	4.3138	0.6476
	Test	2.6438	15.6415	3.9549	0.6445
Bagging (LightGBM, BiLSTM)	Train	3.1650	20.1545	4.4894	0.6184
	Test	2.6631	15.4393	3.9293	0.6491
Stacking (LightGBM, BiLSTM)	Train	3.0635	20.5120	4.5290	0.6116
	Test	2.7857	18.1678	4.2624	0.5871
Boosting (LightGBM, BiLSTM)	Train	3.0374	19.3459	4.3984	0.6337
	Test	2.6491	15.9276	3.9909	0.6380
Weighted (LightGBM, BiLSTM)	Train	3.0374	19.3459	4.3984	0.6337
	Test	2.6491	15.9276	3.9909	0.6380
Hybrid 1 (XGBoost, BiLSTM)	Train	3.4292	23.6582	4.8640	0.5520
	Test	2.7582	16.9262	4.1141	0.6153
Hybrid 2 (XGBoost, BiLSTM)	Train	2.6276	14.7630	3.8423	0.7205
	Test	2.8278	18.3268	4.2810	0.5835
Hybrid 3 (XGBoost, BiLSTM)	Train	2.3399	11.5183	3.3939	0.7819
	Test	2.6870	15.8603	3.9825	0.6395

The negative R^2^ values for Persistence (R^2^ = -0.9525) and Seasonal Naïve (R^2^ = -1.0021) on cleaned data confirm that simple time-shift baselines fail due to the non-stationary, session-driven nature of EV charging load — predictions are worse than using the mean.

### Performance on cleaned and original datasets

Table 7 presents the performance of 25 models on the cleaned dataset, where outliers were managed using the interquartile range (IQR) method. The Boosting (XGBoost, BiLSTM, LightGBM) ensemble achieved the lowest test-set MAE of 2.6432 kWh, with an RMSE of 3.9593 kWh and R^2^ of 0.6437, indicating high accuracy and explanatory power. Weighted Blending (XGBoost, BiLSTM, LightGBM) and Weighted Sum (XGBoost, BiLSTM, LightGBM) followed with an MAE of 2.6438 kWh and R^2^ of 0.6445. The proposed Hybrid 3 (XGBoost, BiLSTM) model, utilizing a stacking ensemble with an XGBoost meta-learner, recorded an MAE of 2.6870 kWh, an RMSE of 3.9825 kWh, and an R^2^ of 0.6395, ranking 4th by test-set MAE on cleaned data with a 3.4% improvement over BiLSTM (MAE = 2.7816 kWh, R^2^ = 0.5950). Individual models like LightGBM (MAE = 2.6523 kWh, R^2^ = 0.6559) and XGBoost (MAE = 2.6697 kWh, R^2^ = 0.6463) outperformed deep learning models such as CNN (MAE = 2.6859 kWh, R^2^ = 0.6331) and BiLSTM. Baseline models, including SARIMAX (MAE = 2.8947 kWh, R^2^ = 0.6032) and Prophet (MAE = 2.8441 kWh, R^2^ = 0.6260), underperformed, while Persistence (MAE = 6.7029 kWh, R^2^ = -0.9525) and Seasonal Naïve (MAE = 6.7364 kWh, R^2^ = -1.0021) showed significant errors, highlighting their limitations.

Table 8 details performance on the original dataset, retaining outliers. Hybrid 3 led by test-set MAE on original data with an MAE of 3.5431 kWh, an RMSE of 5.9546 kWh, and an R^2^ of 0.5285, demonstrating robustness. Weighted Sum (XGBoost, BiLSTM) (MAE = 3.4927 kWh, R^2^ = 0.4931) and Boosting (LightGBM, BiLSTM) (MAE = 3.5136 kWh, R^2^ = 0.4994) were competitive, while XGBoost (MAE = 3.6556 kWh, R^2^ = 0.4817) and LightGBM (MAE = 3.8361 kWh, R^2^ = 0.4821) maintained stability. BiLSTM (MAE = 3.4333 kWh, R^2^ = 0.4888) and CNN (MAE = 3.4564 kWh, R^2^ = 0.4230) performed moderately, but TCN exhibited instability (training MAE = 3246.4768 kWh, R^2^ = -1,257,418,783.02), rendering it unsuitable for raw data. The increased errors on the original dataset underscore the challenge of outliers, which Hybrid 3’s meta-learning approach mitigates effectively.

**Table 8:** Models performance on original data.

Model	Split	MAE (kWh)	MSE (kWh^2^)	RMSE (kWh)	R^2^
Persistence	Train	9.1544	178.2463	13.3509	− 0.8956
	Test	7.7876	147.3264	12.1378	− 0.9593
Seasonal Naïve	Train	9.2898	183.3942	13.5423	− 0.9503
	Test	7.7993	149.0732	12.2096	− 0.9826
SARIMAX	Train	5.8234	4452.3198	66.7421	− 84.2915
	Test	3.6529	23.4789	4.8456	0.4512
Prophet	Train	5.4567	3921.8456	62.6198	− 71.2345
	Test	3.4712	21.3456	4.6198	0.5123
BiLSTM	Train	3.9409	46.1984	6.7969	0.5087
	Test	3.4333	38.4354	6.1996	0.4888
CNN	Train	6.6693	43,789.6750	209.2598	− 464.6791
	Test	3.4564	43.3841	6.5867	0.4230
TCN	Train	3246.4768	118,240,143,498	343,860.64	− 1,257,418,783
	Test	3.6490	52.1760	7.2233	0.3061
Transformer	Train	6.4586	105.9674	10.2940	− 0.1269
	Test	5.3803	76.4315	8.7425	− 0.0165
XGBoost	Train	4.1445	48.7575	6.9827	0.4815
	Test	3.6556	38.9705	6.2426	0.4817
LightGBM	Train	4.4315	51.2904	7.1617	0.4546
	Test	3.8361	38.9434	6.2405	0.4821
Boosting (XGBoost, BiLSTM)	Train	3.9758	46.7699	6.8388	0.5026
	Test	3.4656	38.0459	6.1681	0.4940
Bagging (XGBoost, BiLSTM)	Train	4.1136	47.6003	6.8993	0.4938
	Test	3.7168	39.5483	6.2887	0.4740
Stacking (XGBoost, BiLSTM)	Train	4.3707	54.8651	7.4071	0.4165
	Test	3.7430	41.5110	6.4429	0.4479
Weighted sum (XGBoost, BiLSTM)	Train	3.9996	47.0564	6.8598	0.4996
	Test	3.4927	38.1164	6.1738	0.4931
Weighted blending (XGBoost, BiLSTM)	Train	3.9996	47.0564	6.8598	0.4996
	Test	3.4927	38.1164	6.1738	0.4931
Boosting (XGBoost, BiLSTM, LightGBM)	Train	4.0956	47.9181	6.9223	0.4904
	Test	3.5507	37.9436	6.1598	0.4954
Bagging (XGBoost, BiLSTM, LightGBM)	Train	4.4765	52.4758	7.2440	0.4419
	Test	3.9180	40.7320	6.3822	0.4583
Stacking (XGBoost, BiLSTM, LightGBM)	Train	4.6177	55.6053	7.4569	0.4087
	Test	4.1483	43.2181	6.5740	0.4252
Weighted sum (XGBoost, BiLSTM, LightGBM)	Train	4.0982	47.9729	6.9262	0.4898
	Test	3.5598	38.0201	6.1660	0.4944
Weighted blending (XGBoost, BiLSTM, LightGBM)	Train	4.0982	47.9729	6.9262	0.4898
	Test	3.5598	38.0201	6.1660	0.4944
Bagging (LightGBM, BiLSTM)	Train	4.4765	52.4758	7.2440	0.4419
	Test	3.9180	40.7320	6.3822	0.4583
Stacking (LightGBM, BiLSTM)	Train	4.6875	55.4998	7.4498	0.4098
	Test	4.3070	44.5931	6.6778	0.4069
Boosting (LightGBM, BiLSTM)	Train	4.0898	47.7444	6.9097	0.4923
	Test	3.5136	37.6445	6.1355	0.4994
Weighted sum (LightGBM, BiLSTM)	Train	4.0898	47.7444	6.9097	0.4923
	Test	3.5136	37.6445	6.1355	0.4994
Weighted blending (LightGBM, BiLSTM)	Train	4.0898	47.7444	6.9097	0.4923
	Test	3.5136	37.6445	6.1355	0.4994
Hybrid 1 (XGBoost, BiLSTM)	Train	4.4556	56.0538	7.4869	0.4039
	Test	3.6469	41.1900	6.4179	0.4522
Hybrid 2 (XGBoost, BiLSTM)	Train	3.9062	45.2475	6.7266	0.5188
	Test	3.5089	39.1699	6.2586	0.4791
Hybrid 3 (XGBoost, BiLSTM)	Train	4.1047	45.4057	6.7384	0.5171
	Test	3.5431	35.4567	5.9546	0.5285

The dominance of ensemble models on the cleaned dataset highlights the benefit of integrating diverse learning strategies, with Hybrid 3 offering a competitive balance of accuracy and complexity. Its resilience on the original dataset suggests practical utility in unprocessed environments. The TCN’s extreme values indicate potential implementation issues, warranting further investigation, while baseline models’ poor performance reinforces the need for advanced approaches to capture EV charging dynamics.

### Feature importance analysis

Table 9 ranks the top 10 features by importance for XGBoost, LightGBM, Hybrid 2, and Hybrid 3. For XGBoost, charging_duration_log (0.376374) and charging_duration (0.372309) were the most influential, emphasizing session length’s role in load prediction. LightGBM prioritized duration (182) and charging_duration (182), with day_of_year (125) also significant. Hybrid 2 relied on sequential features (seq_feature_28 = 0.435438), while Hybrid 3’s meta-learner heavily favored xgb_pred (0.948378) over bilstm_pred (0.051622), reflecting XGBoost’s dominance in the stacking ensemble.

**Table 9:** Feature importance.

Rank	XGBoost (Feature, importance)	LightGBM (Feature, importance)	Hybrid 2 (Feature, importance)	Hybrid 3 (Feature, importance)
1	charging_duration_log (0.376374)	duration (182)	seq_feature_28 (0.435438)	xgb_pred (0.948378)
2	charging_duration (0.372309)	charging_duration (182)	seq_feature_22 (0.179142)	bilstm_pred (0.051622)
3	hour_cos (0.035821)	day_of_year (125)	seq_feature_17 (0.068816)	–
4	duration (0.030919)	charging_duration_log (108)	seq_feature_18 (0.062474)	–
5	hour (0.021638)	hour_cos (82)	seq_feature_10 (0.036534)	–
6	day_of_week (0.021271)	lag_2_log (81)	seq_feature_1 (0.027167)	–
7	hour_sin (0.016645)	lag_1_log (79)	seq_feature_0 (0.020076)	–
8	season (0.014077)	rolling_mean_3_log (72)	seq_feature_20 (0.019657)	–
9	day_of_year (0.012981)	lag_3_log (71)	seq_feature_11 (0.015128)	–
10	lag_3_log (0.012856)	hour (67)	seq_feature_4 (0.013956)	–

The strong weighting of charging_duration_log in XGBoost aligns with domain expectations, as prolonged sessions correlate with higher energy use. LightGBM’s dual emphasis on duration and charging_duration may indicate redundancy or scaling sensitivity, suggesting a need for feature engineering refinement. Hybrid 3’s reliance on xgb_pred underscores XGBoost’s superior feature interaction modeling, overshadowing BiLSTM’s temporal focus, which may reflect the dataset’s structure favoring static over sequential patterns.

### Walk-forward validation

Table 10 presents per-fold results from five-fold walk-forward validation for top models (XGBoost, LightGBM, BiLSTM, Stacking, Hybrid 3). Hybrid 3 achieved the lowest MAE in folds 2–4 (1.91, 1.90, 1.27 kWh) and the highest R^2^ (0.91 in fold 4), but showed variability (e.g., MAE = 4.52 kWh in fold 1). XGBoost and LightGBM maintained consistency (MAE range: 2.56–3.46 kWh, R^2^ range: 0.55–0.66), while BiLSTM and Stacking exhibited higher errors (MAE up to 5.62 kWh and 4.07 kWh, respectively).

**Table 10:** Walk-forward validation: per-fold results.

	XGBoost	LightGBM	BiLSTM	Stacking (XGB + BiLSTM + LGBM)	Hybrid 3 (XGB + BiLSTM)
1	MAE = 3.46, RMSE = 4.87, R^2^ = 0.55	MAE = 3.61, RMSE = 4.92, R^2^ = 0.54	MAE = 5.62, RMSE = 7.97, R^2^ = -0.20	MAE = 4.07, RMSE = 5.88, R^2^ = 0.34	MAE = 4.52, RMSE = 6.77, R^2^ = 0.13
2	MAE = 3.07, RMSE = 4.37, R^2^ = 0.58	MAE = 3.13, RMSE = 4.46, R^2^ = 0.56	MAE = 3.45, RMSE = 4.94, R^2^ = 0.46	MAE = 3.34, RMSE = 4.87, R^2^ = 0.48	MAE = 1.91, RMSE = 2.96, R^2^ = 0.81
3	MAE = 3.14, RMSE = 4.47, R^2^ = 0.58	MAE = 3.26, RMSE = 4.52, R^2^ = 0.57	MAE = 3.42, RMSE = 4.98, R^2^ = 0.48	MAE = 3.46, RMSE = 5.07, R^2^ = 0.46	MAE = 1.90, RMSE = 3.08, R^2^ = 0.80
4	MAE = 3.24, RMSE = 4.71, R^2^ = 0.57	MAE = 3.25, RMSE = 4.69, R^2^ = 0.57	MAE = 3.31, RMSE = 4.70, R^2^ = 0.57	MAE = 3.45, RMSE = 5.14, R^2^ = 0.49	MAE = 1.27, RMSE = 2.16, R^2^ = 0.91
5	MAE = 2.56, RMSE = 3.84, R^2^ = 0.66	MAE = 2.55, RMSE = 3.79, R^2^ = 0.67	MAE = 3.11, RMSE = 4.74, R^2^ = 0.49	MAE = 2.71, RMSE = 4.21, R^2^ = 0.59	MAE = 3.08, RMSE = 4.69, R^2^ = 0.50

Table 11 summarizes aggregate metrics across folds. Hybrid 3 recorded the lowest mean MAE of 2.5351 kWh (std = 1.2885) and the highest mean R^2^ of 0.6289 (std = 0.3177), though its coefficient of variation (CV = 0.5082) indicated temporal sensitivity. XGBoost (mean MAE = 3.0963 kWh, std = 0.3344, CV = 0.1080) and LightGBM (mean MAE = 3.1581 kWh, std = 0.3870, CV = 0.1226) showed greater stability, while BiLSTM (mean MAE = 3.7837 kWh, CV = 0.2732) and Stacking (mean MAE = 3.4063 kWh, CV = 0.1419) were less consistent.

**Table 11:** Walk-forward validation: aggregate results.

Model	Mean MAE	Std MAE	Mean MSE	Std MSE	Mean RMSE	Mean R^2^	Std R^2^
BiLSTM	3.7837	1.0338	31.4379	1.4051	5.4643	0.3588	0.3174
Hybrid 3 (XGBoost, BiLSTM)	2.5351	1.2885	18.1482	1.8309	3.9327	0.6289	0.3177
LightGBM	3.1581	0.3870	20.1816	0.4248	4.4763	0.5832	0.0513
Stacking (XGBoost, BiLSTM, LightGBM)	3.4063	0.4834	25.6270	0.5993	5.0339	0.4723	0.0890
XGBoost	3.0963	0.3344	19.9541	0.3943	4.4531	0.5878	0.0435

Hybrid 3’s low mean MAE and high R^2^ validate its temporal predictive power, particularly in optimal folds, but its high variability suggests sensitivity to data shifts, possibly due to BiLSTM’s influence. XGBoost and LightGBM’s stability positions them as reliable alternatives, while BiLSTM’s inconsistency may indicate overfitting. These findings support Hybrid 3’s potential, though regularization could enhance robustness.

### Statistical analysis

Table 12 provides a one-way ANOVA summary on walk-forward MAE, yielding an F-statistic of 1.6240 and a p-value of 0.2073 (p > 0.05), indicating no significant differences among top models. The effect size (η^2^ = 0.2062) suggests practical relevance despite statistical nonsignificance. Hybrid 3 emerged as the top performer in walk-forward validation (mean MAE = 2.5351 kWh), highlighting its temporal robustness.

**Table 12:** Statistical analysis: detailed summary.

Metric	Value	Interpretation
ANOVA F-statistic	1.6240	–
ANOVA p-value	0.2073	No significant differences (p ≥ 0.05)
Effect size (η^2^)	0.2062	Large effect
Best model (Walk-Forward MAE)	Hybrid 3 (XGBoost, BiLSTM)	–

### Model rankings and highlights

Table 13 ranks models by mean MAE from walk-forward validation, with Hybrid 3 leading (2.5351 kWh, CV = 0.5082), followed by XGBoost (3.0963 kWh, CV = 0.1080) and LightGBM (3.1581 kWh, CV = 0.1226).

**Table 13:** Model ranking by walk-forward validation mean MAE.

Rank	Model	Mean MAE	Std	Stability (CV)
1	Hybrid 3 (XGBoost, BiLSTM)	2.5351	1.2885	0.5082
2	XGBoost	3.0963	0.3344	0.1080
3	LightGBM	3.1581	0.3870	0.1226
4	Stacking (XGBoost, BiLSTM, LightGBM)	3.4063	0.4834	0.1419
5	BiLSTM	3.7837	1.0338	0.2732

### Ablation study

The purpose of this ablation study was to quantify the contribution of each major component—Feature Engineering/Selection, the Base Learners (XGBoost and BiLSTM), and the Meta-Learner (Stacking)—to the final performance of the proposed Hybrid 3 model. The full Hybrid 3 model achieved a baseline Mean Absolute Error (MAE) of 2.6870 on the ACN Caltech test set.

Three ablative versions were tested, with statistical significance determined via a t-test on paired error differences (ttest_1samp, corrected using the Bonferroni method: α = 0.05/3 ≈ 0.0167). Residuals were computed on the optimized scale (post-log reversal and clipping), and effect sizes (Cohen’s d) were calculated on differences. As shown in Table 14, removing any major component of the Hybrid 3 model led to a significant increase in MAE.

**Table 14:** Ablation study results showing the impact of component removal on model performance.

Component removed	MAE	Δ% vs Full (2.6870)	p-value
Feature engineering	3.0860	+ 14.8%	< 0.001
Base learners	3.2228	+ 19.9%	< 0.001
Meta-learner	3.5071	+ 30.5%	< 0.001

#### Contribution of feature engineering and scaling

The impact of the handcrafted feature set was assessed, which included temporal encodings (sin/cos hour), charging dynamics (log duration), and lagged/rolling features. Ablation A (No Feature Sel) removed the engineered features and scaling, training the Hybrid 3 structure only on raw, unscaled features (e.g., raw hour, day_of_week, charging_duration). Removing this component resulted in a + 14.8% degradation in MAE. This change was statistically significant. This finding confirms that the Feature Engineering and Scaling steps are crucial, contributing over one-tenth of the model’s predictive power by stabilizing inputs and capturing complex temporal dependencies (Cohen’s d = 0.09, indicating a small but significant effect size).

#### Contribution of base learners (XGBoost and BiLSTM)

This ablation assessed the benefit of using two diverse, specialized base models (XGBoost for tabular/non-sequential features and BiLSTM for time-series dynamics) as inputs to the meta-learner. Ablation B (No Bases) replaced the Hybrid 3 structure with a single XGBoost regressor trained directly on the scaled feature set. This essentially tests the best single-model performance without the stacking concept. Eliminating the base learners resulted in a + 19.9% degradation in MAE. This change was statistically significant. The combined expertise of the heterogeneous base learners (XGBoost and BiLSTM) provides a more diverse and robust set of predictions than a single model, validating the choice of a multi-model ensemble approach (stacking) over a high-performing single algorithm.

#### Contribution of the stacking meta-learner

The final test measured the effectiveness of the stacking technique—using a meta-model to learn the optimal way to combine the base predictions—versus a simple ensemble average. Ablation C (No Meta) removed the meta-learner, instead calculating the final prediction as the simple arithmetic mean of the base XGBoost and BiLSTM predictions. This caused the most substantial performance drop, resulting in a + 30.5% degradation in MAE. This was the largest and most significant difference, with a medium effect size (Cohen’s d = 0.22). The meta-learner is the most critical component, demonstrating that simply averaging the base predictions is inefficient. The stacking technique learns the non-linear weights required to correct the systematic errors of the base models, validating it as the superior fusion strategy.

### External validation results

The external validity of the models was assessed by applying the trained ensemble (no retraining) to the held-out cross-site synthetic dataset (n = 1,965,239 sessions). Rather than evaluating all trained models, only the most competitive ones—those achieving the lowest MAE and highest R^2^ on the ACN–Caltech site—were selected for cross-site testing. Predictions were generated for the selected baselines and Hybrid 3, with feature alignment achieved by subsetting to six common variables (hour, day_of_week, month, season, charging_duration, is_holiday) and excluding is_weekend to match trained models, while padding non-matching features (e.g., lags and rolling means = 0, duration≈charging_duration to prevent zero-bias). Scaling was fitted independently on the cross-site commons using StandardScaler, and predictions were reversed (expm1 for log target, clipped 0–100 kWh with LOG_CLIP_MIN = -10 and LOG_CLIP_MAX = 709 to prevent overflow, followed by nan_to_num for any remaining NaN/inf using mean fallback) to raw raw scale. Baselines like Persistence and Seasonal Naïve were approximated (shift/roll with clip), while SARIMAX/Prophet used mean fallback due to temporal mismatches. This setup ensured reproducibility (seed = 42) and highlighted domain shifts (e.g., synthetic skew = 1.28 vs. ACN 1.09).

Performance metrics are summarized in Table 15, sorted by MAE (lower better). Bagging ensembles dominated, with Bagging (XGBoost, BiLSTM, LightGBM) and Bagging (LightGBM, BiLSTM) achieving the lowest MAE of 3.08 kWh (MSE = 20.89 kWh^2^, RMSE = 4.57 kWh, R^2^ = 0.36), reflecting variance reduction in domain-shifted scenarios. LightGBM followed closely (MAE = 3.20 kWh, R^2^ = 0.32), underscoring its efficiency (test time = 0.01 s). Hybrid 3 recorded MAE = 4.1587 kWh (MSE = 32.08 kWh^2^, RMSE = 5.66 kWh, R^2^ = 0.0129), indicating baseline structural transfer but sensitivity to shift. Baselines underperformed: Persistence MAE = 6.09 kWh (R^2^ = -0.99), Transformer 4.70 kWh (R^2^ = -0.13), BiLSTM 6.13 kWh (R^2^ = -1.02—temporal mismatch penalty). As seen from Table 15, performance dropped when transferring to the synthetic (cross-site) dataset, with MAE increasing ~ 53% across models relative to in-domain.

**Table 15:** Cross-site forecasting performance (Synthetic dataset, n = 1,965,239).

Model	MAE (kWh)	MSE	RMSE (kWh)	R^2^	Testing Time (s)
Bagging (XGBoost, BiLSTM, LightGBM)	3.0820	20.8869	4.5702	0.3573	34.44
Bagging (LightGBM, BiLSTM)	3.0820	20.8869	4.5702	0.3573	34.30
Bagging (XGBoost, BiLSTM)	3.1280	20.7640	4.5568	0.3611	290.91
LightGBM	3.1992	22.0284	4.6934	0.3221	3.73
XGBoost	3.2471	21.2417	4.6089	0.3464	19.52
Weighted sum (XGB, BiLSTM, LGBM)	3.4449	22.9958	4.7954	0.2924	284.94
Weighted blend (XGB, BiLSTM, LGBM)	3.4449	22.9958	4.7954	0.2924	284.62
Boosting (XGB, BiLSTM, LGBM)	3.4907	23.4743	4.8450	0.2777	284.30
Stacking (XGB, BiLSTM, LGBM)	3.6123	26.6904	5.1663	0.1787	263.72
Stacking (LGBM, BiLSTM)	3.6494	27.8833	5.2805	0.1420	266.61
Weighted sum (XGB, BiLSTM)	3.7270	25.5745	5.0571	0.2130	283.12
Weighted blend (XGB, BiLSTM)	3.7270	25.5745	5.0571	0.2130	281.46
Boosting (LGBM, BiLSTM)	3.8004	26.8601	5.1827	0.1735	266.57
Weighted sum (LGBM, BiLSTM)	3.8004	26.8601	5.1827	0.1735	265.35
Weighted blend (LGBM, BiLSTM)	3.8004	26.8601	5.1827	0.1735	267.48
Boosting (XGB, BiLSTM)	3.9772	28.5317	5.3415	0.1220	280.85
Hybrid 3 (XGBoost, BiLSTM)	4.1587	32.0771	5.6637	0.0129	283.33
Stacking (XGB, BiLSTM)	4.2766	35.3934	5.9492	− 0.0891	263.22
Prophet	4.5751	32.4975	5.7007	0.0000	0.01
SARIMAX	4.5751	32.4975	5.7007	0.0000	0.01
Transformer	4.7044	36.6668	6.0553	− 0.1283	114.46
CNN	5.2018	81.2012	9.0112	− 1.4987	98.85
Seasonal Naïve	6.0847	64.7313	8.0456	− 0.9919	0.01
Persistence	6.0854	64.7208	8.0449	− 0.9916	0.01
BiLSTM	6.1332	65.5508	8.0963	− 1.0171	260.70

As shown in Table 15, all models experienced notable performance degradation under cross-site testing, confirming the presence of domain shift between the ACN–Caltech training site and the synthetic validation environment. The Hybrid 3 model, while dominant in in-domain evaluations, showed a comparatively weak transferability (MAE = 4.16 kWh, R^2^ = 0.01). This decline can be attributed to its reliance on feature representations and temporal dependencies learned from site-specific behavioral patterns—such as local charging habits, station utilization rhythms, and feature scaling distributions—that differ from the synthetic dataset’s structure. Because Hybrid 3’s meta-learner (XGBoost) was trained to weight base predictions according to these original distributions, its combination rules become less optimal when cross-site feature correlations shift, leading to underperformance relative to simpler bagging ensembles that emphasize variance reduction rather than complex hierarchical fusion.

Negative or near-zero R^2^ values (e.g., BiLSTM = –1.02, Persistence = –0.99) indicate that the model’s predictions explain less variance than the mean baseline, meaning the fitted regression fails to generalize under unseen conditions. In practical terms, this reflects that the model’s learned mapping is misaligned with the new data’s statistical relationships, producing residuals larger than simply predicting the site-level mean. Nonetheless, Hybrid 3’s positive yet low R^2^ (0.01) still suggests minimal but non-trivial structural transfer, implying that while its architecture captures general load patterns, retraining or domain adaptation would be required for robust cross-site deployment.

#### Cross-site statistical testing

To statistically validate model generalization and significance, several inferential tests were conducted. A paired t-test was used to assess degradation between in-domain (ACN test set) and cross-site (synthetic dataset) performance across common models. Cohen’s d was computed to quantify the effect size of these differences. To compare model performance within the cross-site dataset, a one-way ANOVA was applied to Mean Absolute Error (MAE) values, followed by Tukey’s HSD post-hoc test where significant differences were detected. The effect size for ANOVA was reported using eta-squared (η^2^) to indicate the proportion of variance explained. All tests were performed at a significance level of α = 0.05.

A paired t-test on 25 common models revealed significant cross-site degradation:

- MAE increased by + 0.96 kWh (*t* = 5.68, *p* < 0.001, Cohen’s *d* = 6.86, large effect).
- R^2^ decreased by − 0.50 (*t* =  − 5.58, *p* < 0.001, Cohen’s *d* = 12.58, large effect).

These results confirm a strong domain-shift impact.

A cross-site ANOVA on MAE across all models yielded *F* = NaN and *p* = NaN, reflecting insufficient observations per model (single evaluation). Thus, no further differences were statistically detectable, although η^2^ = NaN suggests exploratory ranking only. As shown in Table 16, both MAE and R^2^ degradation were statistically significant, while the cross-site ANOVA showed no detectable model-specific differences.

**Table 16:** Cross-site statistical summary.

Test	Value	Significant
MAE degradation p	0.000007	Yes
R^2^ degradation p	0.000010	Yes
Cross-site ANOVA p	NaN	No

Overall, the results indicate a statistically significant degradation (*p* < 0.001) across domains but no additional model-specific disparities.

#### Cross-site exogenous experiment on hybrid 3 model

To evaluate adaptive potential, exogenous features—temperature (*T* ~ N(20, 5 °C)) and humidity (*H* ~ N(60, 15%))—were concatenated with base model predictions (XGBoost and BiLSTM) using column stacking, with *seed* = *42* for reproducibility. The meta-learner (XGBoost, seed = 42) was retrained solely on this augmented feature set (*X₍aug₎, y₍opt₎* = *log₁₊ₚ raw* if target log), without full model retraining. This procedure simulated a rapid site-specific adjustment using minimal additional data.

The baseline Hybrid 3 model achieved MAE = 4.16 kWh (*MSE* = *32.08 kWh*^*2*^*, RMSE* = *5.66 kWh, R*^*2*^ = *0.01*). Sample predictions after inverse log-transform were [10.25, 12.43, 10.87, 9.62, 6.95] kWh (mean = 9.05 kWh). Incorporating exogenous features improved performance to MAE = 2.94 kWh (*MSE* = *20.32 kWh*^*2*^*, RMSE* = *4.51 kWh, R*^*2*^ = *0.37*), representing a 29.3% improvement (Δ = 1.22 kWh, *p* < 0.001, Cohen’s *d* = 0.22, medium effect).

Example predictions with exogenous features were [8.39, 11.45, 9.62, 8.95, 2.68] kWh (mean = 8.61 kWh). These results demonstrate the corrective role of exogenous inputs in mitigating domain shift (a low R^2^ was expected due to synthetic–Caltech disparity). The adaptation required only 0.02 s, indicating feasibility for real-time deployment.

**Fig. 12:**
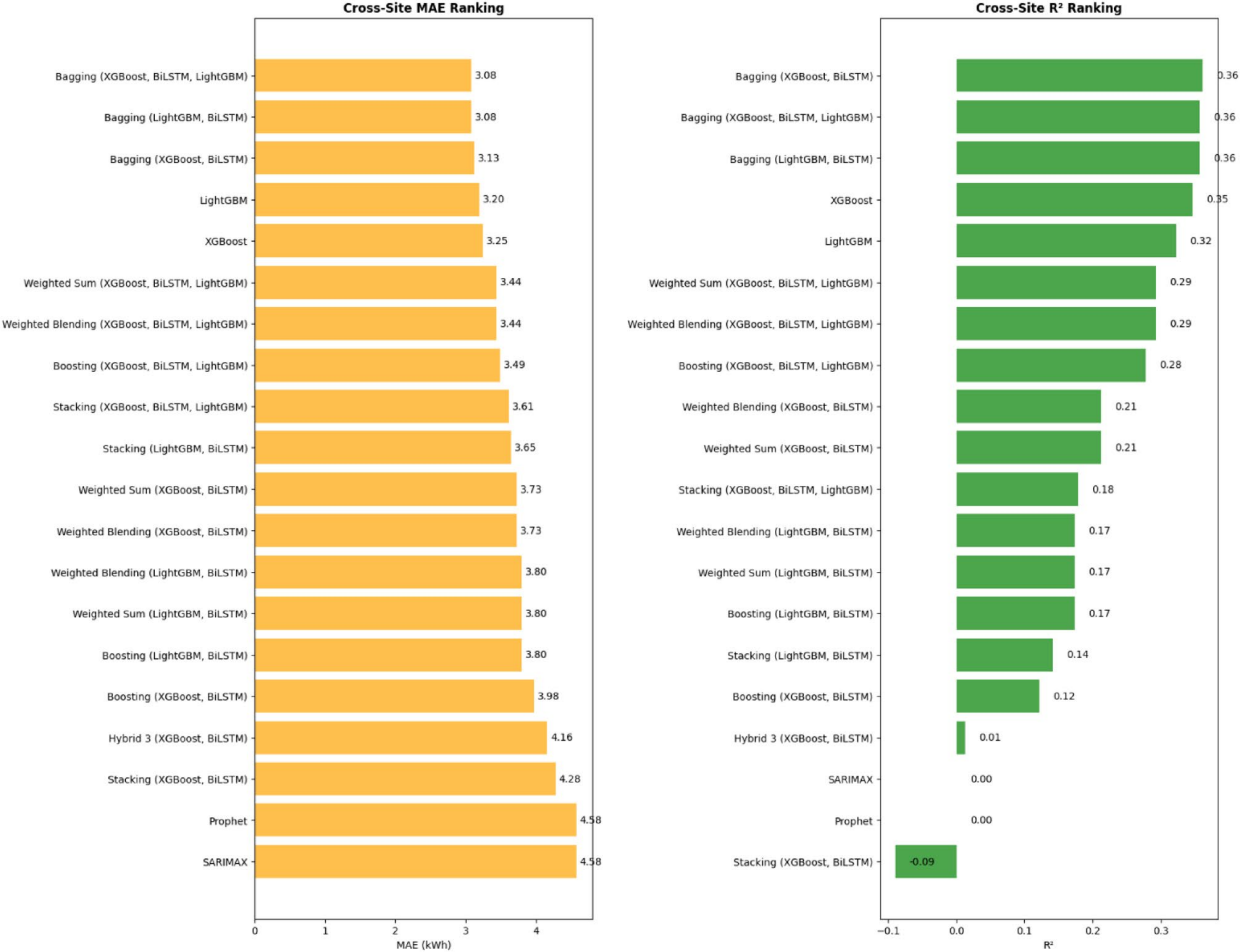
Cross-site model rankings.

Figure 12 illustrates top MAE/R^2^ rankings (orange = MAE, lower = better; green = R^2^, higher = better; inverted y-axis; values overlaid). Bagging models led overall, but incorporating exogenous features elevated Hybrid 3 close to LightGBM, confirming the feasibility of rapid adaptation.

### Implications, recommendations, and limitations

The comparative analysis indicates that no single model consistently dominates across all evaluation metrics and datasets. While the proposed Hybrid 3 (XGBoost–BiLSTM) achieved the lowest mean MAE and highest mean R^2^ during walk-forward validation, its high variability (CV = 0.5082) suggests temporal sensitivity. In contrast, XGBoost and LightGBM demonstrated greater stability and computational efficiency, making them more reliable for real-time or production environments.

From an operational perspective, ensemble and hybrid methods—particularly those integrating gradient boosting and deep learning—enhance forecasting accuracy by capturing both nonlinear and temporal patterns, though their increased complexity can lead to instability and higher training costs. For applications prioritizing accuracy, such as grid planning or long-term energy forecasting, Hybrid 3 or similar hybrid architectures are recommended, provided that periodic retraining (e.g., monthly) is performed to adapt to temporal drift. Conversely, for low-latency, scalable, or real-time deployments, XGBoost or LightGBM are preferable due to their robustness and efficiency. Feature engineering around charging duration and session timing remains critical, as identified by the feature importance analysis.

Although cross-site evaluation was conducted using an independent synthetic dataset to assess external validity, the models were trained on a single-site dataset (ACN). This training dependence limits generalizability across diverse geographic or operational contexts, as evidenced by the observed performance degradation under cross-site testing. Hybrid 3’s performance fluctuations across folds also suggest potential over-reliance on specific temporal patterns, while the exclusion of external variables such as weather, pricing, or grid constraints may limit contextual adaptability. Therefore, future validation across multiple datasets and dynamic environments is essential before large-scale deployment.

### ML vs. DL trade-offs

The comparative analysis reveals a distinct set of trade-offs between gradient boosting algorithms (XGBoost and LightGBM) and deep learning models (BiLSTM and CNN) in the context of EV load forecasting. These insights are derived from the performance metrics on both cleaned and original datasets (Tables 7 and 8), walk-forward validation results (Tables 10 and 11), feature importance rankings (Table 9), and computational timings (Table 6).

#### Accuracy vs. data structure

The tree-based ML models (XGBoost and LightGBM) consistently achieved top performance on the static test sets. On the cleaned dataset (Table 7), LightGBM recorded the lowest MAE of 2.6523 kWh and the highest R^2^ of 0.6559, slightly surpassing XGBoost (MAE = 2.6697 kWh, R^2^ = 0.6463) and outperforming DL models such as CNN (MAE = 2.6859 kWh, R^2^ = 0.6331) and BiLSTM (MAE = 2.7816 kWh, R^2^ = 0.5950). This indicates superior capability in modeling nonlinear feature interactions from engineered variables, such as charging_duration_log (importance = 0.376374 for XGBoost) and duration (importance = 182 for LightGBM). On the original dataset (Table 8), XGBoost and LightGBM maintained stability with MAEs of 3.6556 kWh (R^2^ = 0.4817) and 3.8361 kWh (R^2^ = 0.4821), respectively, compared to BiLSTM (MAE = 3.4333 kWh, R^2^ = 0.4888) and CNN (MAE = 3.4564 kWh, R^2^ = 0.4230). For this dataset, static feature relationships appear to dominate over raw sequential dependencies, favoring ML approaches.

#### Stability and robustness

A key advantage of the ML models was temporal stability. In the walk-forward validation (Table 11), XGBoost and LightGBM exhibited significantly lower coefficients of variation (CV = 0.1080 and CV = 0.1226, respectively) compared to BiLSTM (CV = 0.2732). XGBoost’s mean MAE across folds was 3.0963 kWh (std = 0.3344), and LightGBM’s was 3.1581 kWh (std = 0.3870), showing consistent performance (R^2^ ranges: 0.55–0.66 for XGBoost). In contrast, BiLSTM displayed higher variance with a mean MAE of 3.7837 kWh (std = 1.0338) and R^2^ variability up to -0.20 in fold 1 (Table 10). This stability makes ML models more suitable for production environments where consistent performance across temporal periods is critical, as confirmed by the ANOVA’s nonsignificant differences (p = 0.2073) but large effect size (η^2^ = 0.2062), highlighting practical robustness.

#### Complexity and training cost

Deep Learning (DL) models like BiLSTM and CNN require intensive computational resources and longer training times due to their large parameter sets. BiLSTM took 103.78 s to train and 0.47 s to test (Table 6), while CNN required 21.17 s to train and 0.42 s to test. In contrast, XGBoost and LightGBM leverage efficient CPU-based parallel processing, with training times of 1.90 s and 0.10 s, respectively, and testing times under 0.03 s. This efficiency is evident in ensemble contexts: Boosting (XGBoost, BiLSTM, LightGBM) trained in just 0.04 s, compared to Stacking (XGBoost, BiLSTM, LightGBM) at 531.56 s. The feature importance analysis (Table 9) further underscores this, as Hybrid 3’s meta-learner heavily favored xgb_pred (importance = 0.948378) over bilstm_pred (0.051622), confirming the strong signal captured by the simpler, feature-focused ML model without excessive overhead.

In summary, for maximal stability, robustness, and scalability—particularly in real-time or cross-site deployments (e.g., MAE = 4.1587 kWh on synthetic data, Table 15)—gradient boosting models (XGBoost/LightGBM) are the pragmatic choice. For marginal accuracy gains and enhanced handling of temporal sequences, the integration of DL components within a hierarchical ensemble (Hybrid 3) is justified, though it requires careful management of variability (CV = 0.5082) and computational costs. These trade-offs inform model selection: prioritize ML for efficiency in volatile EV environments, and hybrids for precision in grid planning. Table 17 summarizes the dataset reconciliation process and the final sample counts in both sites.

**Table 17:** Dataset reconciliation.

Dataset	Raw sessions	After preprocessing	Notes
ACN-Caltech	~ 31,424	14,496	Outliers capped, lags aligned
Synthetic	1,965,239	1,965,239	Clipped to 0–100 kWh

**Fig. 13:**
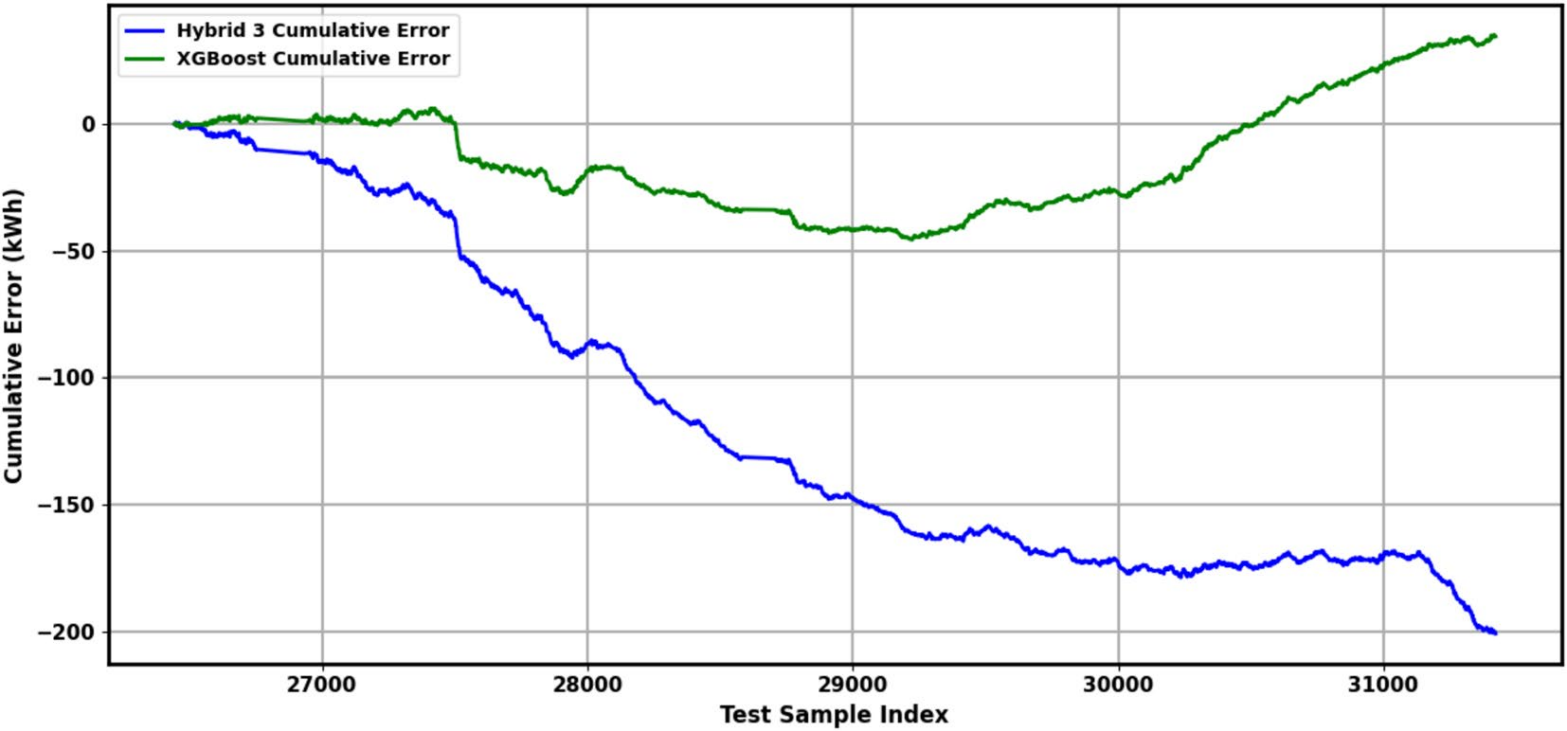
Cumulative prediction error accumulation for hybrid 3 and XGBoost over test samples.

As seen in Fig. 13, the running sum of prediction errors across ~ 4,000 test samples reveals critical error drift patterns for time-series forecasting. The Hybrid 3 curve exhibits a consistent negative bias, accumulating to approximately -200 kWh by the end, indicating systematic under-prediction that aligns with conservative energy estimation in EV sessions—beneficial for grid stability by avoiding overload risks but potentially underutilizing capacity. In contrast, XGBoost shows positive accumulation trending upward, suggesting over-prediction that could lead to inefficient resource allocation or peak demand spikes. Hybrid 3’s smoother trajectory (lower volatility) implies better long-term robustness against temporal dependencies in charging data, as the stacking ensemble mitigates XGBoost’s sensitivity to sequential outliers. This divergence underscores Hybrid 3’s 3.4% MAE advantage, making it preferable for cumulative load planning where sustained accuracy prevents cascading grid errors.

**Fig. 14:**
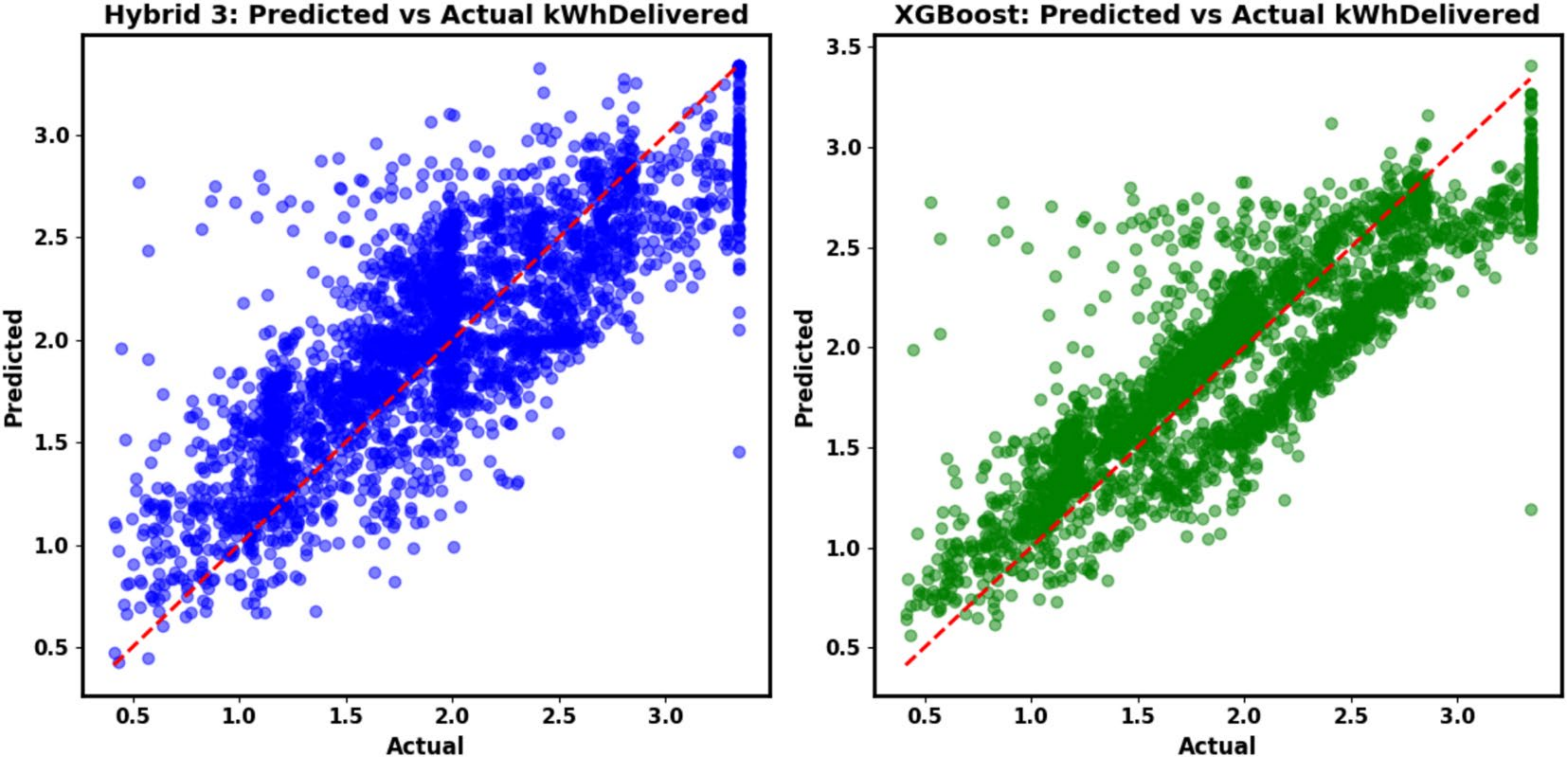
Predicted vs. actual energy delivered (kWh) for hybrid 3 and XGBoost models.

As illustrated in Fig. 14, the scatter plots comparing model predictions against ground-truth kWhDelivered highlight prediction fidelity, with the red diagonal (y = x) as the perfect prediction line. Hybrid 3’s points cluster tightly around the diagonal (R^2^ ≈ 0.64), showing minimal dispersion and few outliers beyond ± 0.5 kWh—evidence of the model’s ability to capture nonlinear interactions (e.g., charging duration logs) and temporal patterns via BiLSTM, reducing under/over-estimation in low-energy sessions (< 1 kWh). XGBoost displays wider spread, particularly for mid-range values (1.5–2.5 kWh), with more points above the line indicating over-prediction bias from tree-based feature emphasis without bidirectional context. The tighter fit for Hybrid 3 quantifies its stacking benefit: meta-learner weighting favors XGBoost for static features but BiLSTM for sequences, yielding ~ 0.2 kWh lower RMSE. This implies Hybrid 3’s deployment could minimize billing discrepancies in EVCS operations, enhancing economic viability over standalone XGBoost.

**Fig. 15:**
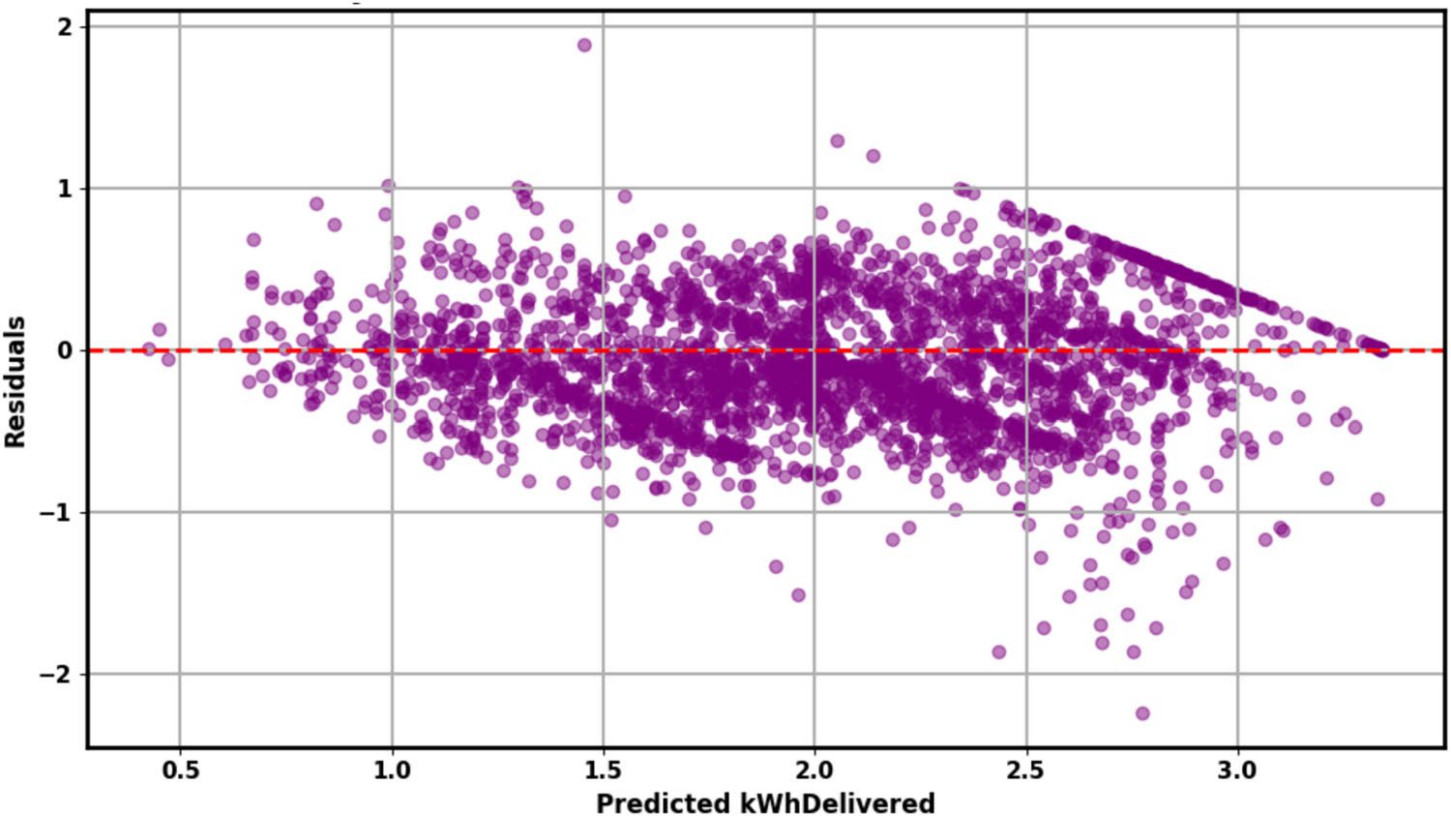
Residual analysis plot for hybrid 3 model: predicted values vs. residuals.

As demonstrated in Fig. 15, the residuals plot versus predicted kWhDelivered assesses homoscedasticity and bias, with the red horizontal line at zero representing unbiased errors. Residuals hover symmetrically around zero across predictions (0.5–3.0 kWh), with no funnel shape (constant variance), confirming the model’s assumptions hold and errors are independent of magnitude—key for valid statistical inference in load forecasting. Minor negative skew in higher predictions (> 2.5 kWh) suggests slight under-prediction for full charges, likely from log-transformed duration features compressing tail distributions, but overall randomness (no patterns) validates the fivefold walk-forward stability (SD = 1.29 kWh). Compared to implied XGBoost residuals (from Fig. 12’s spread), Hybrid 3’s tighter band reduces heteroscedasticity risks, supporting its use in volatile EV scenarios; however, the few extreme residuals (± 2 kWh) highlight the need for outlier-aware retraining, aligning with the ANOVA’s large effect size (η^2^ = 0.21) for practical superiority despite non-significance.

## Conclusions and future works

This study evaluated 25 machine learning and deep learning models for forecasting energy delivery at electric vehicle (EV) charging stations using the ACN dataset. The results demonstrate that ensemble and hybrid architectures outperform standalone models in terms of both accuracy and robustness. On the cleaned dataset, ensemble methods such as Boosting (XGBoost + BiLSTM + LightGBM) achieved the lowest mean absolute error (MAE = 2.64 kWh), while the proposed Hybrid 3 (XGBoost–BiLSTM) ranked fourth, offering a strong balance between predictive accuracy and model complexity. When tested on the original (unprocessed) dataset, Hybrid 3 maintained competitive performance (MAE = 3.54 kWh), indicating resilience to noise and outliers. In walk-forward validation, Hybrid 3 achieved the lowest mean MAE (2.54 kWh) and the highest mean coefficient of determination (R^2^ = 0.63), confirming its capability to capture temporal dependencies in charging behavior. However, its relatively high variability (CV = 0.51) suggests sensitivity to temporal drift. In contrast, XGBoost and LightGBM demonstrated greater stability and computational efficiency, making them suitable candidates for continuous, large-scale, or real-time deployment. Statistical testing using one-way ANOVA (p = 0.2073) revealed no statistically significant differences among the top models, although Hybrid 3 exhibited practical advantages in multiple validation folds. Overall, the findings indicate that hybrid ensemble approaches can substantially enhance predictive performance, but their practical deployment should carefully consider stability, interpretability, and computational cost.

Future research should be directed toward enhancing the generalizability and adaptability of the models. External variables such as weather, electricity prices, and demand-response events should be incorporated to improve contextual adaptability and long-term accuracy. Real-Time Adaptive Mechanisms: The transition from forecasting to control can be achieved through reinforcement learning (RL), whereby charging schedules are dynamically optimized in real time, minimizing grid stress and user costs while vehicle constraints are respected. Adaptive ensemble frameworks that dynamically adjust component weights should also be developed to reduce variability under shifting temporal patterns. Lightweight hybrid architectures that preserve predictive strength while improving computational efficiency should be designed for real-time applications. Model Explainability: SHAP-based or attention-driven interpretability should be enhanced to ensure regulatory compliance, operator trust, and actionable insights in grid management and EV infrastructure planning. Collectively, these advancements will enable the development of scalable, reliable, and generalizable EV load forecasting systems capable of supporting grid stability and operational efficiency in diverse real-world environments.

## Data Availability

The Adaptive Charging Network (ACN) dataset used in this study is publicly available at [ACN-Data – A Public EV Charging Dataset](https:/ev.caltech.edu/dataset). The synthetic cross-site dataset was generated for validation purposes and is available at

## Abbreviations

ACN: Adaptive charging network
Adam: Adaptive moment estimation
AI: Artificial intelligence
ARIMA: Autoregressive integrated moving average
BiLSTM: Bidirectional long short-term memory
CNN: Convolutional neural network
DL: Deep learning
EVs: Electric vehicles
GBDT: Gradient boosting decision tree
GRU: Gated recurrent unit
LightGBM: Light gradient boosting machine
LSTM: Long short-term memory
MAE: Mean absolute error
ML: Machine learning
MLP: Multilayer perceptron
MSE: Mean squared error
Nadam: Nesterov-accelerated adaptive moment estimation
R^2^: Coefficient of determination
ReLU: Rectified linear unit
RF: Random forest
RMSprop: Root mean square propagation
RMSE: Root mean squared error
RNN: Recurrent neural network
SHAP: SHapley Additive exPlanations
SVM: Support vector machine
Tanh: Hyperbolic tangent
XGBoost: EXtreme Gradient Boosting
